# Postprandial lipid and vascular responses following consumption of a commercially-relevant interesterified palmitic acid-rich spread in comparison to functionally-equivalent non-interesterified spread and spreadable butter: a randomised controlled trial in healthy adults†

**DOI:** 10.1039/d3fo05324e

**Published:** 2024-02-15

**Authors:** Wendy L. Hall, Aseel Alkoblan, Philippa S. Gibson, Maria D'Annibale, Astrid Coekaerts, Mathilde Bauer, Johanna H. Bruce, Beryle Lecomte, Armelle Penhoat, Fabienne Laugerette, Marie-Caroline Michalski, Louise J. Salt, Peter J. Wilde, Sarah E. Berry

**Affiliations:** a Department of Nutritional Sciences, School of Life Course and Population Sciences, Faculty of Life Sciences and Medicine, King's College London Franklin Wilkins Building Stamford St. London UK wendy.hall@kcl.ac.uk; b College of Applied Medical Sciences, King Saud bin Abdulaziz University for Health Sciences Riyadh Saudi Arabia; c King Abdullah International Medical Research Center Riyadh Saudi Arabia; d ADM UK Ltd Erith UK; e CarMeN Laboratory INRAE, INSERM U1060, INRAE UMR1397, University of Lyon France; f Food Innovation and Health Programme, Quadram Institute Bioscience UK

## Abstract

*Background*: Interesterification is an industrial processing technique used widely where hard fats are essential for functionality and consumer acceptability, *e.g.* margarines and lower fat spreads. *Objective*: The aim of this study was to compare acute cardiovascular effects of functionally equivalent spreads (similar solid fat content) made with interesterified (IE) or non-IE palm-based fats, or spreadable butter. *Methods*: A randomised, controlled, 4-armed crossover, double-blind study (25 men, 25 women; 35–75 years; healthy; mean BMI 24.5, SD 3.8), compared effects of mixed nutrient meals containing 50 g fat from functionally equivalent products [IE spread, non-IE spread and spreadable butter (SB), with rapeseed oil (RO) as a reference treatment: with 16.7%, 27.9%, 19.3% and 4% palmitic acid, respectively] on 8 h postprandial changes in plasma triacylglycerol (TAG) and endothelial dysfunction (flow-mediated dilatation; FMD). Circulating reactive oxygen species (estimated using a neutrophil oxidative burst assay), glucose, insulin, NEFA, lipoprotein particle profiles, inflammatory markers (glycoprotein acetylation (Glyc-A) and IL-6), and biomarkers of endotoxemia were measured. *Results*: Postprandial plasma TAG concentrations after test meals were similar. However following RO *versus* the 3 spreads, there were significantly higher postprandial apolipoprotein B concentrations, and small HDL and LDL particle concentrations, and lower postprandial extra-large, large, and medium HDL particle concentrations, as well as smaller average HDL and LDL particle sizes. There were no differences following IE compared to the other spreads. Postprandial FMD% did not decrease after high-fat test meals, and there were no differences between treatments. Postprandial serum IL-6 increased similarly after test meals, but RO provoked a greater increase in postprandial concentrations of glycoprotein acetyls (GlycA), as well as 8 h sCD14, an endotoxemia marker. All other postprandial outcomes were not different between treatments. *Conclusions*: In healthy adults, a commercially-available IE-based spread did not evoke a different postprandial triacylglycerol, lipoprotein subclass, oxidative stress, inflammatory or endotoxemic response to functionally-equivalent, but compositionally-distinct alternative spreads. Clinical trial registry number: NCT03438084 (https://ClinicalTrials.gov).

## Abstract 2

*Background*: Interesterification is an industrial processing technique used to create hard fats where this is essential for functionality and consumer acceptability, *e.g.* margarines and lower fat spreads.

## Graphical abstract

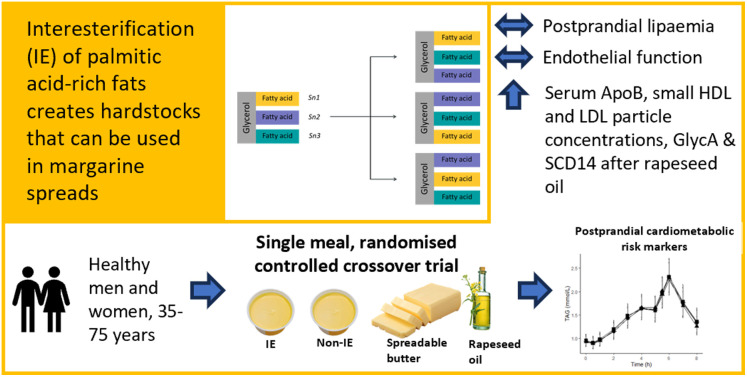

## Introduction

Diets high in saturated fats are linked to an increased risk of cardiovascular diseases (CVD).^1–3^ The main dietary contributors of saturated fatty acids (predominantly palmitic and stearic acid) are animal products such as meats, meat products, and dairy foods, as well as processed foods where formulated fats, which are solid at room temperature, are added for their functional and sensory qualities, mainly cereal-based bakery products, confectionary, and margarines/lower fat spreads.^4,5^ In high-income countries, the role of *trans* fats in processed foods (used for the same functionality) has now been largely replaced by another type of industrially-produced fats, interesterified fats, although recent estimates are that 5 billion people worldwide are still at risk from *trans* fats in the food supply.^6^

Interesterification of fats allows their melting profile and crystallisation properties to be manipulated by altering fat structure, with no change in the overall fatty acid composition.^7^ This involves a chemical or enzymatic catalytic process that results in the rearrangement of fatty acids on the glycerol backbone within and between triacylglycerol molecules, resulting in a greater proportion of saturated fatty acids at the *sn*-2 position and a relative increase in triacylglycerols with saturated fatty acids across all 3 positions (*e.g.* tripalmitate) on the glycerol backbone, which can change the melt profile across temperatures and increase the amount of solid fat present at room temperature.^7^ Blending interesterified fats with other vegetable oils allows production of a range of functional fats with the required amount of solid fat at room temperature and body temperature to yield optimum stability, physical properties during heat processing, and mouthfeel. By using interesterified fats instead of their native equivalents or animal-sourced hard fats, the overall saturated fatty acid content of the food product can be lowered.^7,8^ The most common IE fats available in European markets up to now are from blends of palm oil fractions, and therefore rich in palmitic acid (16 : 0).^5^ Prior to interesterification, fractionation of fats can be used to separate solid and soft fats from their original native fat, *e.g.* palm oil can be fractionated into palm olein and palm stearin. If interesterification were not used by the oils and fats industry, hard fats would need to contain a greater proportion of saturated fatty acids and different proportions of fats (*e.g.* lauric acid rich fats such as coconut oil and palm kernel oil) in order to achieve a spread with equivalent sensory and functional properties.^5^

Although the adverse effects of saturated and *trans* fats on lipids and other cardiovascular risk factors are well-documented, there is a lack of clarity on the cardiometabolic health impact of interesterified fats. Several studies have suggested that some interesterified fats can modify postprandial lipaemia^9–14^ compared to non-interesterified equivalents. Historically, increased postprandial lipaemia was attributed to the *sn*-2 hypothesis which suggested that saturated fatty acids from interesterified fats are absorbed more quickly because interesterification leads to a greater proportion of 2-monoacylglycerols with a saturated fatty acid and a lower proportion of saturated fatty acids in the *sn*-1 and *sn*-3 positions. Saturated fatty acids in the outer positions of triacylglycerol molecules are hydrolysed preferentially during digestion and therefore absorbed less easily as free fatty acids due to their high individual melting point, as well as being more likely to be excreted, as reviewed by Mills *et al.*^11^ It is now known that the determinants of whether interesterification increases or reduces postprandial lipaemia depends on the individual fat blends that are being interesterified and the resultant changes in the solid fat content of the fat blend at body temperature. In fact, commonly-used palm oil-based interesterified hardstocks (hardstocks being the solid fat that is blended with other oils to make spreads or cooking/baking fats) do not modify postprandial triacylglycerol concentrations or lipoprotein particle sizes/concentrations in healthy 45–75 years old adults compared to a non-interesterified hardstock with an identical fatty acid profile and similar melt profile.^15^

The effects of palm-based interesterified fats on endothelial function have not yet been investigated. Palmitic acid decreases nitric oxide availability and induces endothelial cell inflammation *in vitro*.^16^ Circulating palmitic acid has been associated with inflammation^17^ and impaired endothelial function,^18^ although this may reflect a link between endogenous palmitic acid synthesis and inflammation. Few clinical trials have investigated whether there is a direct causal relationship between dietary palmitic acid intake and inflammation or endothelial function,^19^ and these suggest no adverse effects on inflammatory markers.^20,21^ There is a lack of research on the effects of commercially relevant IE palmitic acid-rich fats on postprandial lipaemia or related cardiovascular risk markers when consumed as the final fat product rather than the original hardstocks.

This research aims to compare the acute cardiometabolic effects of a commercially available interesterified fat spread (IE) with functional comparators (similar melt profile): (1) non-interesterified alternative spread with a differing fatty acid profile (non-IE), and (2) spreadable butter (SB). A reference liquid oil (rapeseed; RO) was included to aid cross-comparison with previous studies that have included MUFA-rich oils. Primary outcomes were: (1) endothelial function measured by flow-mediated dilatation, and (2) postprandial lipaemic responses, measured by incremental area under the curve for plasma triacylglycerol concentrations. Secondary outcomes included serum lipoprotein particle profiles, neutrophil NADPH oxidase activity, serum inflammatory markers Glyc-A and IL-6, biomarkers of endotoxemia, plasma glucose, and serum insulin and NEFA. We hypothesised that a test meal containing IE fat spread would induce a similar degree of postprandial lipaemia relative to functional comparators due to their similar solid fat content at body temperature,^8,12^ but that IE would lead to a reduced postprandial impairment in endothelial function due to its lower palmitic acid content. We tested this hypothesis in a randomised controlled crossover trial in equal numbers of healthy men and women: the Inter-Cardio study. This work is complemented by assessment of *in vitro* rates of lipolysis of test fats to elucidate whether there were any differences in lipid digestion and lipase action.

## Methods

### Subjects

Ethical approval for the Inter-Cardio study was obtained from King's College London Research Ethics Committee (ref. HR-17/18-5499) and written informed consent was provided by participants. The study was conducted in accordance with the ethical standards laid down in the 1964 Declaration of Helsinki and its later amendments. The trial was registered at clinicaltrials.gov as NCT03438084.

Participants were recruited *via* advertisements at King's College London and the surrounding area through distribution of flyers, email circulars, social media and newspaper advertisements between April 2018 and July 2019. Inclusion criteria were males and females aged 35–75 years, healthy (free of diagnosed diseases specified in exclusion criteria), able to understand the information sheet and willing to comply with study protocol, and able to give informed consent. Exclusion criteria were medical history of myocardial infarction, angina, thrombosis, stroke, cancer, liver or bowel disease or diabetes; body mass index < 20 kg m^−2^ or >35 kg m^−2^; plasma cholesterol ≥7.5 mmol L^−1^; plasma triacylglycerol (TAG) > 3 mmol L^−1^; plasma glucose > 7 mmol L^−1^; blood pressure (BP) ≥ 140/90 mmHg; current use of antihypertensive or lipid lowering medications; alcohol intake exceeding 28 units per week; current cigarette smoker (or quit within the last 6 months); active blood donor or plans to donate blood within 6 months of study completion; and ≥20% 10-year risk of CVD as calculated using a risk calculator.

Respondents to advertisements were sent a participant information sheet and an initial screening questionnaire and, if deemed eligible, invited in for a screening visit at the Department of Nutritional Sciences, King's College London following an overnight fast. Their weight, height, waist and hip circumference, percentage body fat, seated clinic blood pressure, haematology, glucose, liver function and lipid profile were assessed to confirm no underlying medical conditions that would make them ineligible to take part. Participants were requested to complete a 3-day diet diary to assess their habitual nutritional intakes at baseline.

### Study design

**Fig. 1:**
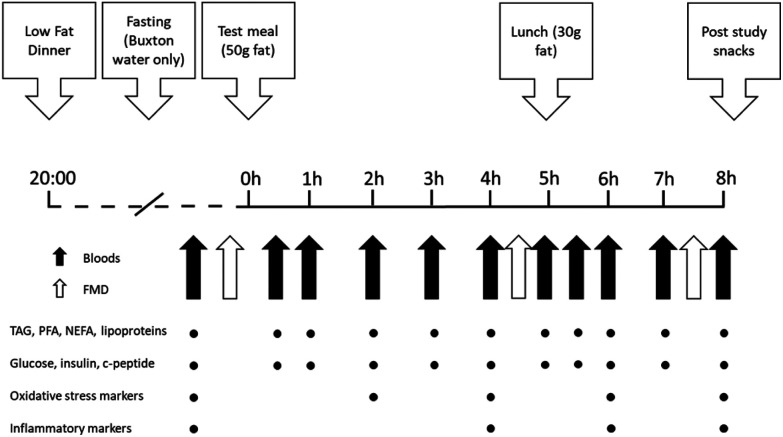
Study design. FMD, flow-mediated dilatation; TAG, triacylglycerol; PFA, plasma fatty acids; NEFA, non-esterified fatty acids.

A randomised, controlled, double-blind, 4-phase crossover design was used to compare 50 g test fat with a minimum 1-week washout period (Fig. 1). Treatment sequence was randomised using online software (https://www.randomization.com) by a trial investigator. Muffins containing the test fats were allocated codes by a technician independent of the study, with both investigators and participants blinded to the identity of treatments. For 24 h prior to study days, participants were asked to refrain from the following: consumption of caffeine, alcohol, high-fat, high-sugar, high-salt foods, nuts, and nitrate-rich foods, and to abstain from vigorous exercise. Participants were required to consume a low fat (<20 g) standardised evening meal at least 12 hours before the time of the study day appointment, and to only drink low-nitrate mineral water (Buxton; Nestlé) thereafter. Study day appointments occurred between 07:30 and 09:00. Upon arrival, weight and body composition was analysed using bioelectrical impedance scales (Tanita™ Body Composition Analyser), a cannula was positioned in the antecubital vein of the forearm, and baseline blood samples were taken. Next participants rested in a supine position in a quiet, temperature-controlled room for 15 min before baseline measures of endothelial function were taken using flow-mediated dilatation (FMD), as previously reported.^22^ After all baseline measures were made, test meals were consumed within a 15 min time window, and then further blood samples were taken at 30, 60, 120, 180, 240, 300, 330, 360, 420 and 480 min, and further FMD measurements were taken at 270 and 450 min. After 300 min, a metabolic challenge second meal was consumed; this was identical across all 4 arms.

### Power calculation

Based on our group's previously published data and guidelines for FMD measurement^23,24^ it was calculated that an estimated sample size of 20 males and 20 females would allow detection of a difference in means for % FMD of 2.16 (SD 2.9),^24^ with 90% power (alpha 0.05), for both sexes separately. A postprandial difference of 2% in FMD is within the clinically significant range and shown to be associated with CVD risk.^25^ To allow for potential attrition of 20%, 50 was set as the target for recruitment (25 men and 25 women). This would also allow detection of a mean difference of 1.2 mmol L^−1^ h^−1^ (iAUC) in postprandial plasma TAG (SD 1.8 ^15^) for both sexes separately, or a mean difference of 0.73 mmol L^−1^ h^−1^ in the whole sample population (men and women combined) with 80% power (alpha 0.05),^9^ clinically meaningful differences that have been shown to be predictive of risk of CVD.^26^

### Test meals

**Fig. 2:**
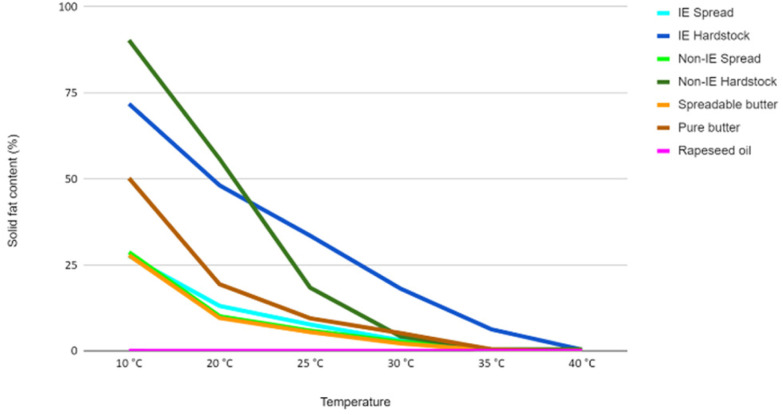
Solid fat content of test fats at temperatures 10–40 °C. Solid fat contents of spreads and their respective hardstocks (in the case of spreadable butter, compared to the pure, unblended butter), prior to blending with rapeseed oil, at temperatures from 10 to 40 °C. All fats contained 5% or less solid fat content at body temperature. IE, interesterified.

Each test meal consisted of two muffins (made with test spreads) and a milkshake and provided 3.75 MJ (897 kcal), 16 g protein (7% energy), 88 g carbohydrate (39% energy), and 50 g test fat (54% energy); all meals were similar in appearance and taste. The 4 test fats were as follows: a commercially available spread containing IE fat, a non-IE spread formulated to match the IE spread in melt profile (created by Johanna Bruce, ADM Fats & Oils), a commercially available spreadable butter, and reference liquid oil, refined rapeseed oil (Sainsburys PLC) (see Table 1 for fatty acid composition and Fig. 2 for melt characteristics). Both the IE spread and the spreadable butter are readily available in all UK supermarkets and convenience stores. The proportions of lauric acid (12 : 0), palmitic acid (16 : 0) and stearic acid (18 : 0) in the 4 test spreads, measured by gas chromatography, were as follows: IE, 10.2, 16.7 and 2.5%; non-IE, 4.1, 27.9 and 3.5%; B, 2.0, 19.0 and 8.5%; RO, 0.0, 4.0 and 1.5%. Percentage solid fat contents at 10 °C and close to body temperature (35 °C), measured by NMR (European Laboratories of ADM Hamburg AG), were as follows: IE, 27.6 and 0%; non-IE, 28.7 and 0.4%; B, 27.8 and 0.1%; RO is a liquid oil so had no solid fat content. The test spreads/oil were baked into muffins, labelled with a code by a technician not involved in the study, and stored frozen until consumption. To mimic a more real-life scenario, a standardised metabolic challenge test meal was provided at 5 h postprandially as an additional oral fat challenge, containing a total of 30 g of fat from rapeseed oil, 15 g protein and 55 g carbohydrates in the form of a muffin. Rapeseed oil was chosen as it has previously been shown to induce a pronounced postprandial lipemic response that is not impacted by the presence of solid fat,^9^ and the meal was standardised across all treatment arms to ensure that any differential responses post-timepoint 5 h reflected the effect of the initial test meal. All test muffin recipes contained cornstarch, pastry flour, caster sugar, baking powder, egg white powder, skimmed milk, vanilla essence, and the appropriate amount of test fat to provide 50 g fat per muffin.

**Table 1:** Fatty acid composition of the experimental fats (weight%)

Fatty acid	IE^a^	Non-IE^b^	Spreadable butter^c^	Rapeseed oil
12 : 0	10.2	4.1	2.0	0
14 : 0	3.8	1.9	8.0	0.1
16 : 0	16.7	27.9	19.3	4.0
18 : 0	2.5	3.5	8.5	1.5
**Total saturated fatty acids**	**33.2**	**37.4**	**37.8**	**5.6**
18 : 1n-9 *cis*	44.5	44.3	37.8	61.5
18 : 1n-9 *trans*	0	0	2.0	0
18 : 2n-6 *cis*	13.3	11.7	8.2	20.0
18 : 2n-6 *trans*	0	0.1	0.1	0
18 : 3n-3 *cis*	5.6	4.0	3.7	10.0
18 : 3n-3 *trans*	0	0.3	0	0
18 : 2n-6 *cis* : 18 : 3n-3 *cis*	2.4	2.9	2.2	2.0
**Total unsaturated fatty acids**	**63.4**	**60.4**	**51.8**	**91.5**

a IE (interesterified) spread: 65% fat (76.9 g added per test meal to provide 50 g fat) from palm oil fractions (conventional palm oil, palm stearine) 23%, palm kernel oil 22%, rapeseed oil 55%. Spread contains 20% buttermilk.

b Non-IE spread: 65% fat (76.9 g added per test meal to provide 50 g fat) from palm oil (mid fraction) 45%, rapeseed oil 40%, palm kernel oil 15%. Buttermilk added at 20% of final spread to match IE spread.

c Spreadable butter: 78% fat (64.1 g added per test meal to provide 50 g fat) made with 74% butter blended with 26% rapeseed oil. Data are presented as % of total weight.

### Dietary analysis

Baseline diets were characterised using 3-day diet diaries. Participants were instructed to record non-weighed portions of all meals, snacks and drinks consumed (except water), for a period of 3 days, including 1 weekend day.

### Flow-mediated dilatation

Endothelial function was assessed by flow-mediated dilatation of the brachial artery according to standard guidelines^23^ as previously reported^22^ using a Vivid iq ultrasound unit with a 12 MHz transducer (GE Healthcare, Buckinghamshire, UK) and cuff inflation to 200 mmHg for 5 min. Images were captured at 60 seconds after cuff deflation during reactive hyperaemia to record maximum artery diameter dilation. Images were analysed using automatic edge-detection software (Brachial Analyser, Medical Imaging Applications, Iowa City, US) by a single researcher blinded to treatment allocation, and quality checked by an independent technician. Before each postprandial measurement, participants rested in a supine position for 10 min. % FMD was calculated as the % change in post-occlusion diameter from the pre-occlusion diameter. Intra-observer CVs for repeat measurements within the same day were 3.8–7.7%.

### Blood processing

Blood samples taken during the screening visit were analysed by an accredited clinical pathology laboratory (Affinity Biomarker Laboratories, London, UK).

Study visit blood samples were collected into BD Vacutainer® SST™ tubes for analysis of serum TAG, non-esterified fatty acids (NEFA), IL-6, insulin, and lipoprotein particle analysis; BD Vacutainer® heparin tubes for analysis of neutrophil NADPH oxidase activity, and BD Vacutainer® Fluoride/Oxalate tubes for analysis of plasma glucose concentrations. SST™ and Fluoride/Oxalate tubes were centrifuged at 1300*g* 4 °C for 15 min, and serum and plasma samples were stored at −70 °C pending analysis. Plasma glucose, and serum insulin and NEFA concentrations were analysed at 0, 30, 60, 120, 180, 240, 300, 330, 360, 420 and 480 min; serum IL-6 was analysed at 0, 240, 360 and 480 min; serum lipoproteins, % MUFA, PUFA and SFA, and GlycA were analysed at 0, 120, 240, 360 and 480 min; serum TAG was analysed at 0, 60, 120, 180, 240, 300, 330, 360, 420 and 480 min; neutrophil NADPH oxidase activity was analysed at 0, 240, and 360 hours; and endotoxemia biomarkers were analysed in plasma at 0, 240 and 480 min on each study.

Serum/plasma TAG (Randox Cat. No. FA115), glucose (Werfen Cat. No. 00018250840) and NEFA (WAKO NEFA-HR Cat. No. 434-91795/436-91995) concentrations were analyzed using enzymatic colorimetric assays on an ILAB-650 clinical chemistry analyzer (Instrumentation Laboratories™); replicate measure CVs were <2%, <2% and <3% respectively. Serum insulin and c-peptide (Siemens Healthcare Diagnostics Ltd, Frimley, Surrey, UK) and high sensitivity IL-6 (V-PLEX human IL-6 kit, Meso Scale Discovery, Rockville, MD 20850, USA) were analysed by ELISA by Affinity Biomarker Laboratories, interassay CVs less than 6%, 3%, and 9% respectively. Serum lipoprotein profiles (size and particle number), apolipoprotein (Apo) B, ApoA1, fatty acid composition (% omega-3 and -6, SFA, MUFA and PUFA) and inflammatory marker, GlycA, were quantified by high throughput proton NMR by Nightingale Health (Nightingale Health Ltd, Helsinki, Finland).^23,27^

### Neutrophil NADPH oxidase activity

NADPH oxidase-derived superoxide (a reactive oxygen species) can inactivate endothelium-derived nitric oxide, linking it with endothelial dysfunction.^28^ Neutrophil NADPH oxidase activity was measured by the neutrophil oxidative burst assay and flow cytometry. Full details of the methods are in ESI.†

### Endotoxin biomarkers

Lipopolysaccharide (LPS)-binding protein (LBP, an LPS transporter) and soluble cluster of differentiation 14 (sCD14, a glycoprotein expressed on the surface of monocytes and macrophages and a soluble receptor that receives LPS from LBP, resulting in endothelial cell activation), and the ratio between them, are used as more stable markers of endotoxin exposure than direct measurement of LPS,^29^ and have been shown previously to be modified postprandially following high fat meals.^30,31^ Plasma LBP and sCD14 concentrations were assayed using ELISA kits (CliniSciences and R&D Systems; Nanterre, France) as described elsewhere.^32^

### *In vitro* lipolysis

To determine whether the composition of the different test fats affected susceptibility to hydrolysis by lipase, *in vitro* lipolysis experiments were performed on each product, as validated previously.^33^ To normalise the effect of the structure of each product and ensure that the fat substrates were equally accessible, the fat phases were homogenised to form model oil in water emulsions prior to *in vitro* lipolysis.

The approach used was the same as for our previous study.^15^ Briefly, the aqueous phase used was a 1.0% solution of whey protein isolate (WPI) (Supplied by Davisco Foods International Inc. (Minnesota, USA)) in ultrapure water. The test materials (spreads and RO) were heated to 70 °C to melt the fats to aid emulsification. A 5.0 wt% oil in water emulsion was prepared by mixing 5.0 g of the melted fat phase with 95.0 g of the 1% WPI solution using a Silverson L4R Laboratory homogeniser at 7600 rpm for 5 minutes (5 × 1 minute with a 30 s interval between each period). The particle size distribution of each emulsion was determined using a laser diffraction size analyzer (LS13320 Beckman Coulter, Indianapolis, IN, USA). Results confirmed that each emulsion had a monomodal size distribution (ESI Fig. 1†), and all had a similar mean droplet diameter (*D*_4,3_) of approximately 3 μm. The emulsion sample (4.0 mL) was mixed with the simulated intestinal fluid [100 mM PBS, 10 mM bovine bile, 0.6 mM CaCl_2_(H_2_O)_2_], the pH was adjusted to 7.0 using 0.1 mM NaOH and finally, porcine pancreatin (Sigma-Aldrich product code P7545) was added to give a final concentration of 100 U mL^−1^ in a final volume of 40 mL digestion mixture. Samples were digested for 2.0 h with constant stirring (700 rpm) at 37 °C, using a KEM AT-700 titrator, (Kyoto Electronics, GPS Instrumentation Ltd, Clifford, UK) to maintain the pH at 7.0 using 0.1 M NaOH. The amount of 0.1 M NaOH titrated over time was used to determine % lipolysis, which was calculated using the following formula:
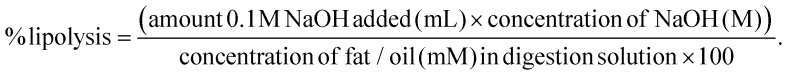


### Statistical analysis

Statistical analysis was performed using IBM SPSS Statistics version 28.0.1.1. For each of the analyses described below, the assumptions of normality and homogeneity of variance were assessed, and natural log or square root transformation carried out where necessary. For GlycA and IL-6, a normal distribution was not achieved following transformation and analysis was conducted using Schwarz's Bayesian criterion to assess the best fitting model. To eliminate the possibility of signal interference in the NMR analysis from triacylglycerol or MUFA peaks, additional analyses were conducted on untransformed data using GlycA to serum VLDL diameter ratio or GlycA to serum MUFA% ratio. Incremental AUC (iAUC) was calculated using the trapezoidal rule, including iAUC (0–8 h) and iAUC (0–4 h) for TAG, glucose, insulin, c-peptide and NEFA. *C*_max_ (maximum concentration) and *T*_max_ (time of maximum concentration) was calculated for TAG only. Incremental AUC, *C*_max_, and time point data following each treatment were analyzed using a linear mixed model. For the calculation of iAUC, if one value was missing over the assessment period, the iAUC was generated by imputation using the average of the values available either side of the missing time point. If there was >1 missing value or either of the start or end values were missing, no iAUC was calculated for that visit for that participant. Terms in the model included treatment group, time point (except for iAUC and *C*_max_), sex, treatment × sex, period, and treatment × period as fixed effects (and treatment × time for time point analysis), participant ID as a random effect, and baseline value as covariate. Comparisons of baseline (pre-test meal) outcome measures were also made by linear mixed model including treatment group, sex, period, treatment × sex, treatment × period as fixed effects and participant ID as a random effect. Participants who did not complete all 4 study visits or where there were missing data at individual time points but data from at least one of the study periods or other time points in all study periods were included in the analysis by including participant as a random effect in the analysis. *Post hoc* analyses were made using Bonferroni adjustment for multiple comparisons. *T*_max_ was treated as a categorical variable and analysed by Chi square test. For all tests, the significance level was set at *P* < 0.05 (2-tailed).

## Results

### Participant characteristics

**Fig. 3:**
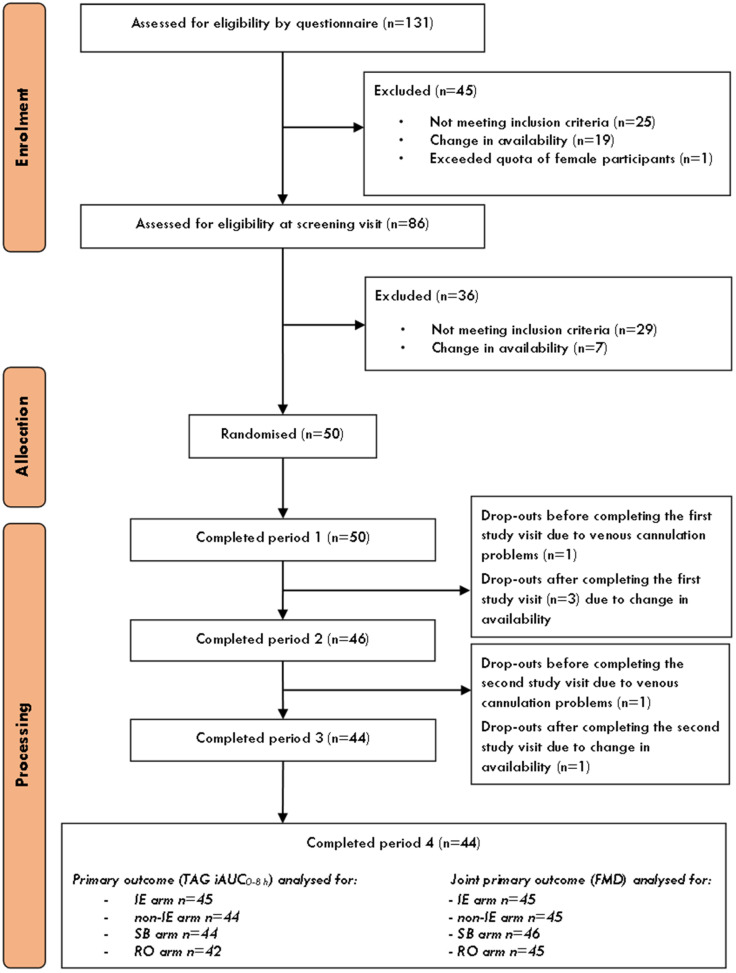
Flow diagram of the progress through the phases of the trial. FMD, flow-mediated dilatation; iAUC, incremental area under the curve; IE, interesterified; RO, rapeseed oil; SB, spreadable butter; TAG, triacylglycerol.

A total of 131 volunteers were assessed for eligibility for participation by the initial questionnaire (Fig. 3). At this point, 45 volunteers were excluded (25 did not meet inclusion criteria, 19 were no longer available, and 1 female was not needed as there were sufficient females already recruited); 86 were further assessed for eligibility at a clinic screening visit, of whom 50 remained eligible and available so were randomised to treatment sequence (Fig. 3). One participant dropped out prior to the first study visit, and 3 after the first study visit. A further 2 participants dropped out after the second study visit leaving a total of 44 participants completing all 4 study visits. Baseline characteristics of the randomised men (*n* = 25) and women (*n* = 25) are listed in Table 2. Of the female participants, 17 were postmenopausal. Mean age was 54.7 years (±11.9) and mean BMI and median serum TAG were 24.5 kg m^−2^ (±3.8) and 0.8 mmol L^−1^ (±0.41) respectively.

**Table 2:** Baseline characteristics of enrolled trial participants

	Total^*b*^	Females^*c*^ (*n* = 25)	Males^*c*^ (*n* = 25)
Age, years	54.7 ± 11.0	56.2 ± 10.2	53.7 ± 11.6
BMI, kg m^−2^	24.5 ± 3.8	25.5 ± 4.5	23.3 ± 2.5
Waist circumference, cm	86.7 ± 11.3	85.8 ± 13.6	87.1 ± 8.4
% body fat	26.2 ± 10.2	34.4 ± 6.7^*d*^	17.9 ± 5.7
Systolic BP, mmHg	112.4 ± 10.7	110.4 ± 13.3	114.1 ± 7.0
Diastolic BP, mmHg	75.1 ± 7.4	74.4 ± 8.6	75.8 ± 6.0
Plasma glucose^*a*^, mmol L^−1^	5.1 (0.5)	5.1 (0.5)	5.2 (0.5)
Serum total cholesterol, mmol L^−1^	5.3 ± 0.9	5.4 ± 0.9	5.1 ± 0.9
Serum LDL cholesterol, mmol L^−1^	3.2 ± 0.8	3.2 ± 0.7	3.2 ± 0.9
Serum HDL cholesterol, mmol L^−1^	1.8 ± 0.5	1.9 ± 0.5	1.8 ± 0.4
Serum triacylglycerol^*a*^, mmol L^−1^	0.8 (0.4)	0.8 (0.5)	0.9 (0.4)
Total: HDL cholesterol	3.0 ± 0.8	3.0 ± 0.8	3.1 ± 0.9
Habitual energy/macronutrient intake			
Energy^*a*^, MJ	7.6 (2.1)	6.8 (1.8)^*d*^	8.3 (2.2)
Fibre^*a*^ (AOAC), g	22.7 (8.7)	22.7 (8.7)	22.8 (9.0)
Carbohydrate^*a*^, g	197.8 (61.7)	179.2 (50.4)^*d*^	216.4 (68.7)
Carbohydrate^*a*^, % energy	44.6 (8.8)	45.3 (9.8)	43.9 (8.1)
Free sugars^*a*^, % energy	5.3 (4.1)	4.9 (4.0)	5.6 (4.3)
Fat^*a*^, g	72.3 (30.1)	65.8 (33.1)	78.7 (26.8)
Fat^*a*^, % energy	35.2 (8.1)	35.4 (9.7)	35.1 (6.5)
Saturated fatty acids^*a*^, % energy	11.1 (3.5)	10.9 (3.9)	11.4 (3.3)
Protein^*a*^, g	77.7 (25.5)	68.8 (17.6)^*d*^	86.7 (29.8)
Protein^*a*^, % energy	17.2 (3.0)	17.1 (2.9)	17.4 (3.3)

### Serum fatty acid composition

**Fig. 4:**
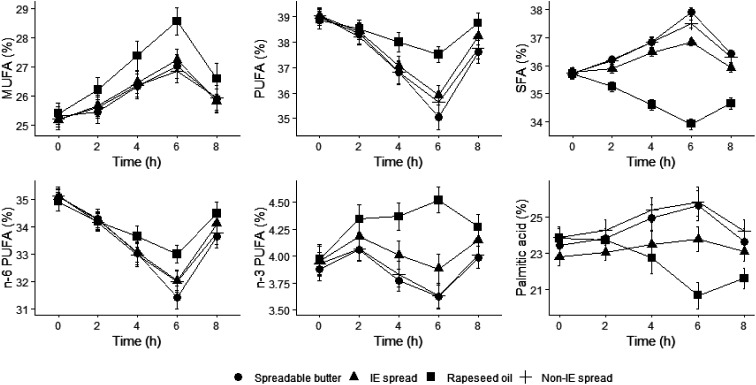
Postprandial serum fatty acid concentrations following a commercially available interesterified (IE) spread, a functionally equivalent non-IE spread, and spreadable butter, relative to a reference rapeseed oil (RO). Serum total MUFA, PUFA, SFA, n-6 PUFA, n-3 PUFA, and palmitic acid as percentages of total serum fatty acids (mol%). Data are mean (SEM), *n* = 44–46. Treatment, time and treatment × time interaction effects were all highly statistically significant *P* < 0.001. MUFA, PUFA, SFA, n-6 PUFA, n-3 PUFA analysed by NMR spectroscopy and palmitic acid by GC. MUFA, monounsaturated; PUFA, polyunsaturated; SFA, saturated.

The main fatty acid classes and palmitic acid are presented in Fig. 4 to demonstrate the postprandial changes that occurred over time following consumption of the test fats. Postprandial changes reflected the fatty acid composition of the meals. Serum MUFA, PUFA, SFA, n-6 PUFA, and n-3 PUFA as mol% and palmitic acid as weight% showed significant treatment effects and treatment × time effects for all (*P* < 0.001).

### Postprandial lipemia

**Fig. 5:**
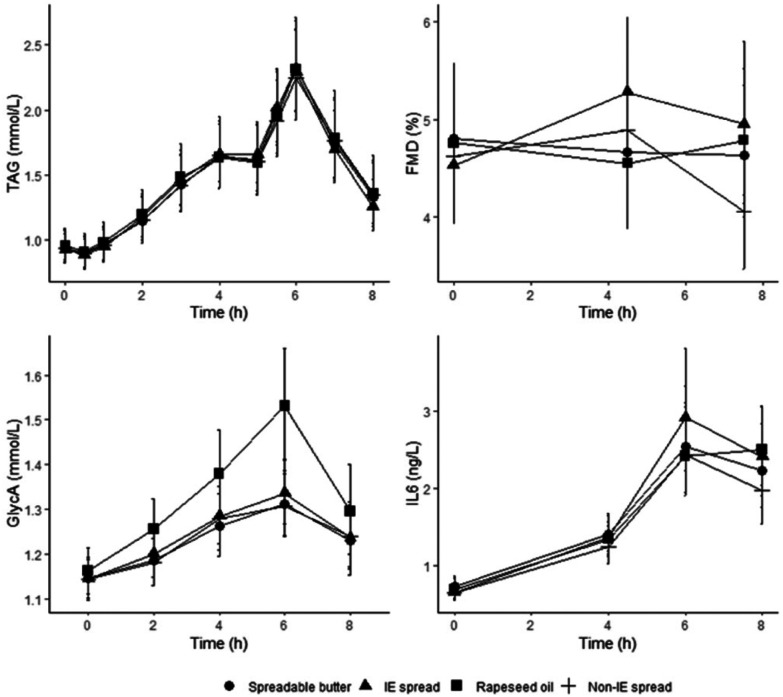
Postprandial serum triacylglycerol (TAG) concentrations, flow-mediated dilatation (FMD), and serum GlycA and interleukin-6 (IL-6) concentrations following a commercially available interesterified (IE) spread, a functionally equivalent non-IE spread, and spreadable butter, relative to a reference rapeseed oil (RO). Serum TAG (*n* = 45) concentrations, FMD (*n* = 46), and serum GlycA (*n* = 45) and IL-6 (*n* = 45) concentrations following a test meal containing 50 g fat from 3 different spreads (one commercially available containing IE palm oil fractions, palm kernel oil and RO; one, a non-IE equivalent from mid-fraction palm oil, palm kernel oil, and RO; and the other a spreadable butter (made with butter and RO) relative to a reference RO). Data are geometric means with 95% confidence intervals. Comparison of test fats by linear mixed-model analysis (dependent variable postprandial values, fixed factors of treatment, time, period, treatment × time interaction, treatment × period interaction; random effect participant; covariate baseline, and sex where significant) showed no significant treatment or treatment × time effects for plasma TAG, FMD and IL-6. Significant treatment effect for GlycA (*P* < 0.001) and treatment × time interaction for GlycA (*P* < 0.001). * Bonferroni-adjusted *post hoc* pairwise comparisons showed that postprandial GlycA concentrations were significantly higher following RO consumption compared to IE (mean difference 0.082 mmol L^−1^, 95% CI 0.048, 0.116), non-IE (mean difference 0.092 mmol L^−1^, 95% CI 0.058, 0.126) and SB (mean difference 0.095 mmol L^−1^, 95% CI 0.061, 0.129); all *P* < 0.001. There were significant time effects for TAG, GlycA, and IL-6 (*P* < 0.001).

There were no differences in postprandial serum TAG concentrations between treatments (Fig. 5), as assessed by iAUC (0–8 h), iAUC (0–4 h), *C*_max_ and *T*_max_ (Table 3). There was a significant sex effect for iAUC (0–4 h): mean 1.26 mmol L^−1^ h^−1^ (0.94, 1.70) in women and 0.75 mmol L^−1^ h^−1^ (0.55, 1.05) in men; the treatment × sex interaction was not significant. There were no period effects or treatment × period interactions for iAUC (0–8 h), iAUC (0–4 h), *C*_max_ and *T*_max_. At baseline, there were no significant differences in serum TAG concentrations between study visits by period or treatment. Across all treatments and timepoints there was a significant effect of time due to increased postprandial TAG concentrations following meals (*P* < 0.001; Fig. 5), but no significant treatment × time interactions.

**Table 3:** Postprandial incremental area under the curve, peak concentration and time of peak concentrations for plasma triacylglycerol concentrations following an interesterified (IE) spread, non-IE spread, spreadable butter, and a reference rapeseed oil (RO)

	RO (reference)	IE spread (IE)	Non-IE spread (non-IE)	Spreadable butter (SB)	Overall treatment effect (*P* value)	Overall sex effect (*P* value)	Treatment × sex effect (*P* value)
iAUC (0–8 h), mmol L^−1^ h^−1^	4.60 (3.79, 5.57)	4.44 (3.75, 5.27)	4.38 (3.67, 5.22)	4.16 (3.30, 5.25)	0.831	0.327	0.699
iAUC (0–4 h), mmol L^−1^ h^−1^	1.23 (1.00, 1.52)	0.82 (0.55, 1.24)	1.02 (0.79, 1.30)	0.86 (0.61, 1.21)	0.032^a^	0.018^b^	0.242
*C*_max_, mmol L^−1^	2.44 (2.21, 2.70)	2.42 (2.22, 2.64)	2.43 (2.21, 2.66)	2.43 (2.21, 2.67)	0.997	0.935	0.496
*T*_max_, h	6 (6, 6)	6 (5.5, 6)	6 (5.5, 6)	6 (5.5, 6)	0.239^c^	NA	NA

a Pairwise comparisons between treatments revealed no significant differences (Bonferroni adjusted).

b Mean iAUC (0–4 h) was 1.26 mmol L^−1^ h^−1^ (0.94, 1.70) in women and 0.75 mmol L^−1^ h^−1^ (0.55, 1.05) in men; mean difference between sexes 23.6% (9.9, 158.8); data were natural log transformed before analysis, mean differences generated as log ratios and converted to a percentage.

c Pearson Chi square test (*n* = 45).

### Endothelial function

There were no significant treatment effects, treatment × time interactions, time effects, sex effects, treatment × sex interactions, period effects, or treatment × period interactions for FMD (Table 4 and Fig. 5). There were no treatment effects, treatment × time interactions, time effects, period effects, or treatment × period interactions for pre-occlusion brachial artery diameter, nor peak brachial artery diameter (Table 4). As expected, there were significant sex effects (*P* < 0.05) for pre-occlusion/peak brachial artery diameters (Table 4), with diameters being significantly greater in males than females. However, there were no significant treatment × sex interactions for brachial artery diameters. There were no differences between baseline values between arms.

**Table 4:** Postprandial flow-mediated dilatation, brachial artery diameters, and NADPH oxidase activity following an interesterified (IE) spread, non-IE spread (non-IE), spreadable butter (SB), and a reference rapeseed oil (RO)

	Test meal	Baseline	4.5 h (4 h for NADPH oxidase)	7.5 h (7 h for NADPH oxidase)	Treatment effect	Time effect	Treatment × time effect	Sex effect	Treatment × sex effect
FMD^a^, %	*RO*	*4.55 (4.33, 4.79)*	*4.52 (3.86, 5.30)*	*4.75 (4.16, 5.43)*	0.175	0.728	0.361	0.182	1.00
	IE	4.68 (4.50, 4.87)	5.30 (4.63, 6.07)	5.01 (4.33, 5.80)					
	Non-IE	4.70 (4.52, 4.88)	4.89 (4.21, 5.70)	4.06 (3.52, 4.68)					
	SB	4.71 (4.54, 4.89)	4.58 (3.98, 5.28)	4.55 (3.96, 5.22)					
Pre-occlusion brachial artery diameter, mm	*RO*	*3.80 (3.66, 3.95)*	*3.83 (3.77, 3.88)*	*3.84 (3.79, 3.90)*	0.539	0.362	0.844	0.034	0.722
	IE	3.83 (3.72, 3.93)	3.79 (3.73, 3.84)	3.81 (3.76, 3.87)					
	Non-IE	3.80 (3.69, 3.91)	3.79 (3.74, 3.85)	3.83 (3.77, 3.90)					
	SB	3.77 (3.68, 3.87)	3.84 (3.79, 3.90)	3.83 (3.76, 3.90)					
Peak brachial artery diameter, mm	RO	3.99 (3.86, 4.14)	4.08 (3.94, 4.22)	4.03 (3.96, 4.11)	0.524	0.808	0.772	0.014	0.964
	IE	4.01 (3.87, 4.15)	4.01 (3.95, 4.07)	4.04 (3.97, 4.10)					
	Non-IE	4.00 (3.86, 4.14)	4.00 (3.94, 4.06)	4.00 (3.94, 4.07)					
	SB	3.97 (3.83, 4.12)	4.03 (3.97, 4.09)	4.01 (3.94, 4.09)					
NADPH oxidase activity index^b^^,^^c^	RO	514.8 (435.1, 601.3)	287.2 (237.0, 342.2)	249.8 (205.8, 298.1)	0.052	0.024	0.897	0.286	0.242
	IE	435.8 (352.9, 527.3)	306.5 (242.8, 377.6)	279.9 (217.4, 350.3)					
	Non-IE	488.3 (400.8, 584.4)	345.4 (291.1, 404.4)	309.1 (266.7, 354.6)					
	SB	525.0 (444.5, 612.1)	305.8 (242.2, 376.9)	242.7 (195.4, 295.1)					

a Geometric means with 95% CI. There were no significant differences between treatment arms at baseline. Postprandial data (4.5 h and 7.5 h) analysed on an intention-to-treat basis using a linear mixed model (fixed factors treatment, sex, period, treatment × sex, treatment × period; baseline as covariate; participant ID as a random factor).

b Data square root transformed before analysis by linear mixed model.

c Reduced sample size due to technical failure during analysis or sample loss, *n* = 41.

### Apolipoproteins and lipoprotein particles

**Fig. 6:**
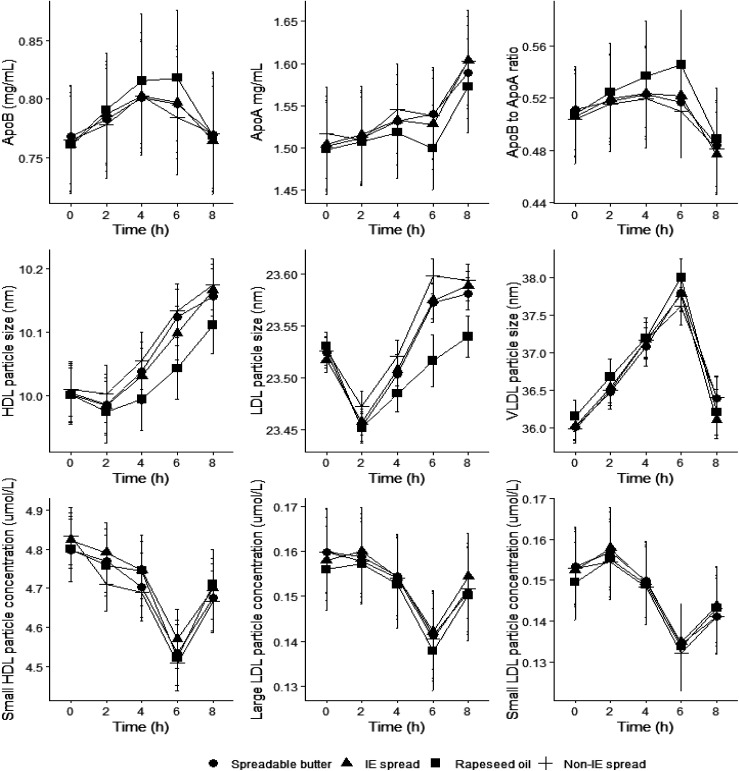
Postprandial serum apolipoprotein concentrations, lipoprotein particle sizes, and lipoprotein subclass particle concentrations following a commercially available interesterified (IE) spread, a functionally equivalent non-IE spread, and spreadable butter, relative to a reference rapeseed oil (RO). Serum apolipoprotein and lipoprotein concentrations, and average lipoprotein particle sizes (*n* = 44–46) following a test meal containing 50 g fat from 3 different spreads (one commercially available containing IE palm oil fractions, palm kernel oil and RO; one, a non-IE equivalent from mid-fraction palm oil, palm kernel oil, and RO; and the other, a spreadable butter made with butter and RO) relative to a reference RO. Data are geometric means with 95% confidence intervals for ApoB, ApoA1, ApoB : ApoA1 ratio, large LDL particle concentrations, and small LDL particle concentrations, and means with standard errors for average HDL, LDL and VLDL particle sizes and small HDL particle concentrations. Comparison of test fats by linear mixed-model analysis (dependent variable postprandial values; fixed factors of treatment, time, period, treatment × time interaction, treatment × period interaction; random effect participant; covariate baseline) showed significant treatment effects for ApoB (*P* < 0.001), ApoA1 (*P* < 0.05), ApoB : ApoA1 ratio (*P* < 0.001), average HDL particle size (*P* < 0.001), average LDL particle size (*P* < 0.001), small HDL particle concentration (*P* < 0.05), large LDL particle concentrations (*P* < 0.05), and small LDL particle concentrations (*P* = 0.001). ApoA1, apolipoprotein A1; ApoB, apolipoprotein B; HDL, high-density lipoprotein; LDL, low-density lipoprotein; VLDL, very low-density lipoprotein.

Postprandial serum apolipoprotein B, apolipoprotein A1 and lipoprotein subfraction particle concentrations and average sizes are presented in ESI Table 1.† All the apolipoprotein (apo) and lipoprotein parameters significantly changed during the postprandial period irrespective of treatment (Fig. 6). There were significant treatment effects for ApoB (*P* < 0.001), ApoA1 (*P* < 0.05), ApoB : ApoA1 ratio (*P* < 0.001), concentrations of extra-large to small HDL particle concentrations and average HDL particle size (*P* < 0.05–0.001), and small and large LDL particle concentrations and average LDL particle size (*P* < 0.05–0.001) (Fig. 6), but there were no significant treatment effects on VLDL parameters. *Post hoc* pairwise comparisons (Bonferroni-adjusted) showed that ApoB concentrations were significantly higher postprandially following RO compared with non-IE (*P* < 0.001) and SB (*P* = 0.001), ApoA1 was significantly lower following RO compared with IE (*P* < 0.05) and SB (*P* < 0.05), and ApoB : ApoA1 ratio was significantly higher postprandially following RO compared with IE (*P* < 0.001), non-IE (*P* < 0.001) and SB (*P* < 0.001).

Extra-large and large HDL particle concentrations were significantly lower postprandially following RO compared with IE (*P* < 0.001), non-IE (*P* < 0.001) and SB (*P* = 0.005 and <0.001, respectively), and M-HDL particle concentrations were significantly lower postprandially following RO compared with IE (*P* < 0.005) and SB (*P* < 0.05). S-HDL particle concentrations were significantly higher postprandially following RO compared with non-IE (*P* < 0.05). *Post hoc* Bonferroni-adjusted pairwise comparisons were not statistically significant between treatments for L-LDL particle concentrations. S-LDL particle concentrations were significantly higher following RO compared with non-IE (*P* < 0.005) and SB (*P* < 0.05). Bonferroni-adjusted pairwise comparisons showed that the significant treatment effect for HDL and LDL particle sizes were due to reductions following RO compared to IE (mean differences: HDL, −0.036 nm, 95% CI −0.047, −0.024, *P* < 0.001; LDL, −0.039 nm, 95% CI −0.052, −0.026, *P* < 0.001), non-IE (HDL, −0.042 nm, 95% CI −0.054, −0.031, *P* < 0.001; LDL, −0.043 nm, 95% CI −0.055, −0.030, *P* < 0.001), and SB (HDL, −0.035 nm, 95% CI −0.046, −0.023, *P* < 0.001; LDL, −0.030 nm, 95% CI −0.042, −0.017), and reductions in average LDL particle sizes following SB compared with non-IE (−0.013 nm, 95% CI −0.026, −0.001, *P* < 0.05).

### NADPH oxidase activity

There were no significant differences in neutrophil NADPH oxidase activity indices at baseline between treatment arms. Across all treatment arms there was a significant time effect (*P* < 0.001) but no significant treatment × time interaction, indicating a significant decrease in NADPH oxidase activity following meals that did not differ according to treatment (Table 4). There was no significant treatment effect, indicating no postprandial differences in neutrophil superoxide production between spreads and RO.

### Inflammatory markers

There were no significant differences between treatment arms for baseline GlycA and IL-6 values prior to test meals being consumed. Both GlycA and IL-6 significantly increased postprandially (time effect *P* < 0.001). There were no significant treatment differences in postprandial IL-6 concentrations, nor treatment × time interactions. However, there was a significant treatment effect on GlycA (*P* < 0.001; Fig. 5) and a significant treatment × time interaction (*P* < 0.001); Bonferroni-adjusted *post hoc* pairwise comparisons showed that postprandial GlycA concentrations were significantly higher following RO consumption compared to IE (mean difference 0.082 mmol L^−1^, 95% CI 0.048, 0.116), non-IE (mean difference 0.092 mmol L^−1^, 95% CI 0.058, 0.126) and SB (mean difference 0.095 mmol L^−1^, 95% CI 0.061, 0.129); all *P* < 0.001. Adjustment for possible interference MUFA peaks in the NMR analysis was conducted for GlycA using GlycA : MUFA% ratio; this did not change the outcome.

### Endotoxaemia markers

**Fig. 7:**
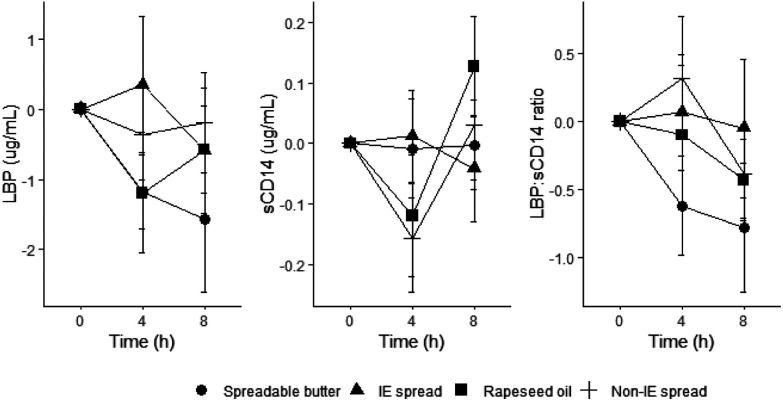
Postprandial plasma concentrations of endotoxin biomarkers following a commercially available interesterified (IE) spread, a functionally equivalent non-IE spread, and spreadable butter, relative to a reference rapeseed oil (RO). Changes from baseline in plasma lipopolysaccharide-binding protein (LBP) concentrations, soluble cluster of differentiation 14 (sCD14) concentrations, and LBP : sCD14 ratio (*n* = 31–40 per treatment/time point) concentrations following a test meal containing 50 g fat from 3 different spreads (one commercially available containing interesterified (IE) palm oil fractions, palm kernel oil and rapeseed oil; one a non-IE equivalent from mid-fraction palm oil, palm kernel oil, and rapeseed oil; and the other a spreadable butter made with butter and rapeseed oil) relative to a reference rapeseed oil. Data are means with standard errors. Comparison of test fats by linear mixed-model analysis (dependent variable postprandial values, fixed factors of treatment, time, period, treatment × time interaction, treatment × period interaction; random effect participant; covariate baseline) showed no significant treatment or time effects. There was a treatment × time interaction (*P* = 0.031) and a significant sex effect (*P* = 0.031) for sCD14; there was a tendency for sCD14 to decrease postprandially in males and increase in females.

There were no significant differences between treatment arms for baseline concentrations of plasma LBP, sCD14, or LBP/SCD14 ratio prior to test meals being consumed. There were significant time effects for sCD14, indicating postprandial effects of high-fat meals on this biomarker of endotoxin exposure, but not LBP or LBP/SCD14 ratio (Fig. 7). Regarding overall treatment differences, there were significant treatment × time interactions for sCD14 across 4 and 8 h (*P* < 0.05) (Fig. 7) but *post hoc* comparisons did not reveal any significant treatment differences at any specific timepoint. There was a significant sex effect (*P* = 0.031) for sCD14, with sCD14 decreasing postprandially in males and slightly increasing in females (ESI Fig. 2†). Finally, there were no period effects or treatment × period interactions.

### Glucose, insulin, C-peptide and NEFA

**Fig. 8:**
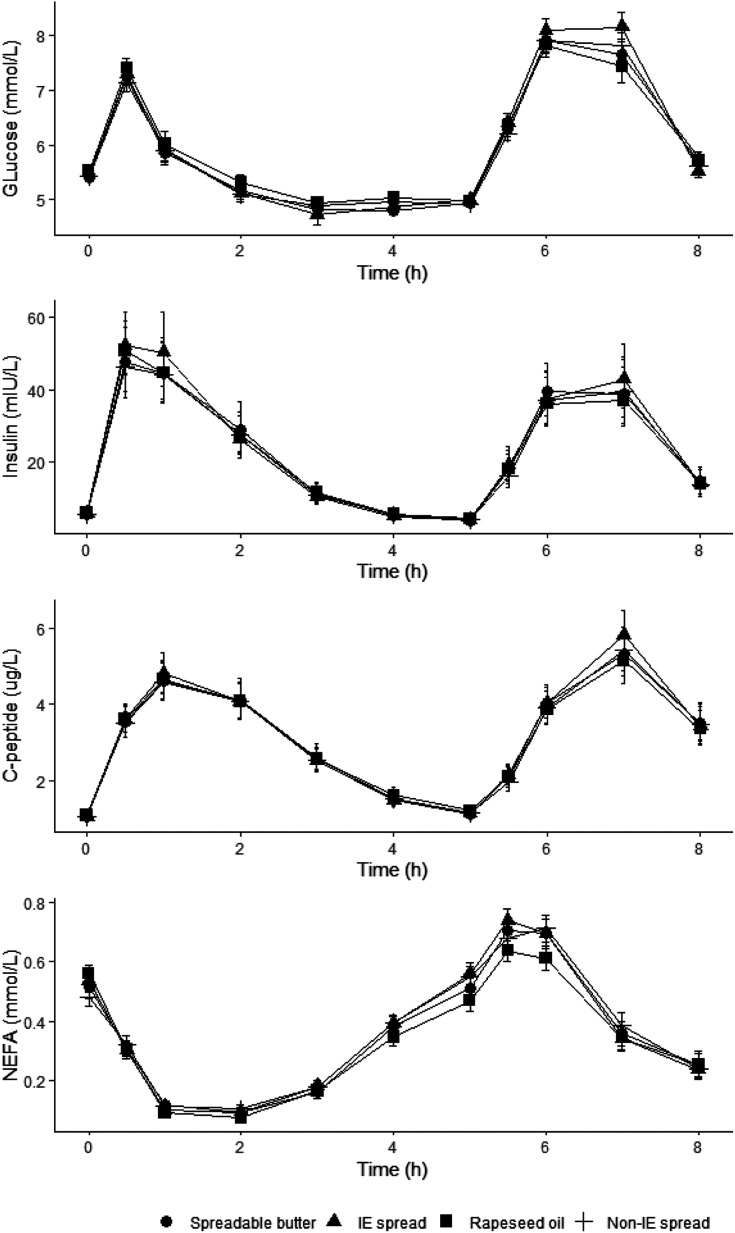
Postprandial plasma glucose concentrations, and serum insulin, C-peptide and non-esterified fatty acid (NEFA) concentrations following a commercially available interesterified (IE) spread, a functionally equivalent non-IE spread, and spreadable butter, relative to a reference rapeseed oil (RO). Mean (SE) plasma glucose concentrations (*n* = 44–45 per treatment/time point), geometric mean (95% CI) serum insulin and c-peptide concentrations (*n* = 43–45 per treatment/time point), and mean (SE) serum NEFA concentrations (*n* = 44–45 per treatment/time point) following a test meal containing 50 g fat from 3 different spreads (one commercially available containing interesterified (IE) palm oil fractions, palm kernel oil and rapeseed oil; one a non-IE equivalent from mid-fraction palm oil, palm kernel oil, and rapeseed oil; and the other a spreadable butter made with butter and rapeseed oil) relative to a reference rapeseed oil. Comparison of test fats by linear mixed-model analysis (dependent variable postprandial values, fixed factors of treatment, time, period, treatment × time interaction, treatment × period interaction; random effect participant; covariate baseline) showed significant time effects (*P* < 0.001 for all) but no significant treatment effects or treatment × time interactions for glucose, insulin and C-peptide. Significant treatment effects for NEFA iAUC (0–4 h) and iAUC (0–8 h) (*P* = 0.014 and *P* = 0.002 respectively) were observed; *post hoc* tests with Bonferroni adjustment showed that the decrease in NEFA up to 4 hours was significantly greater following RO compared to non-IE (mean difference −0.13 mmol L^−1^ h^−1^, 95% CI −0.25, −0.02, *P* = 0.015), and up to 8 hours was significantly greater following TO compared to SB, IE and non-IE (mean difference RO-SB was −0.29 mmol L^−1^ h^−1^, 95% CI −0.57, −0.00, *P* = 0.048, mean difference RO-IE was −0.30 mmol L^−1^ h^−1^, 95% CI −0.54, −0.06, *P* = 0.008, and mean difference RO-non-IE was −0.41 mmol L^−1^ h^−1^, 95% CI −0.69, −0.13, *P* = 0.001).

Plasma glucose and serum insulin, c-peptide and NEFA all changed significantly over time (*P* < 0.001). There were no differences in baseline concentrations prior to test meals between treatments. No effect of treatment was found for glucose, insulin and C-peptide iAUC (0–4 h), iAUC (0–8 h) (Fig. 8; ESI Table 2†). There were significant treatment effects for NEFA iAUC (0–4 h) and iAUC (0–8 h) (*P* = 0.014 and *P* = 0.002 respectively); *post hoc* tests with Bonferroni adjustment showed that the decrease in NEFA up to 4 hours was significantly greater following RO compared to non-IE (mean difference −0.13 mmol L^−1^ h^−1^, 95% CI −0.25, −0.02, *P* = 0.015), and up to 8 hours the iAUC was significantly smaller following RO compared to SB, IE and non-IE (mean difference RO-SB was −0.29 mmol L^−1^ h^−1^, 95% CI −0.57, −0.00, *P* = 0.048, mean difference RO-IE was −0.30 mmol L^−1^ h^−1^, 95% CI −0.54, −0.06, *P* = 0.008, and mean difference RO-non-IE was −0.41 mmol L^−1^ h^−1^, 95% CI −0.69, −0.13, *P* = 0.001).

### *In vitro* lipolysis rates

Exploration of lipolysis curves (ESI Fig. 3†) show that the emulsions made with the spreads initially digested at a similar rate: IE achieved 25% lipolysis in 9 min, non-IE in 7 min, and SB in 6 min. By 120 min, lipolysis achieved was 38%, 41%, and 40% for IE, non-IE and SB respectively. The RO emulsion was significantly slower to digest reaching 25% lipolysis in a much longer time of 28 min and only achieving 33% lipolysis after 120 min. Lipolysis rates cannot be attributed to the droplet size distribution of the emulsions (ESI Fig. 1†), which were similar for all products.

## Discussion

The present study investigated the postprandial lipid and vascular responses of healthy adults following the consumption of commercially-available margarine-type spreads made with interesterified (IE) palm-based fats compared to non-interesterified (non-IE) functionally-equivalent spreads. The objective was to assess potential cardiovascular risk effects associated with interesterification, a widely used processing technique in the fats and oils industry, and, for the first time, to assess this in the final products that are consumed in real life, rather than assessing effects of the hardstocks (which are blended with liquid oils to form the spreads) that have been used as test fats in our previous studies. We also set out to investigate a wider range of novel postprandial cardiovascular risk markers, including arterial flow-mediated dilatation, biomarkers of endotoxin exposure and oxidative stress, and inflammatory markers.

Our previous study, using hardstocks, showed that interesterified and non-interesterified palm-based fats with equivalent fatty acid composition and similar melt profiles had equivalent effects on *in vitro* fat digestion, and *in vivo* postprandial TAG responses and lipoprotein profiles,^15^ suggesting that interesterification did not modify intestinal digestion and lipid metabolism. Consequently, in the present study it was hypothesized that the IE fat spread would induce a similar degree of postprandial lipaemia relative to the non-IE functionally-equivalent (but differing in fatty acid composition) spread and spreadable butter due to their similar solid fat content at body temperature, regardless of differences in fatty acid composition. As hypothesized, despite the differences in fatty acid composition, postprandial lipemic responses were not significantly different between the test meals containing the spreads (IE, non-IE, and SB); nor compared to rapeseed oil (RO), a liquid reference oil, suggesting that solid fat content (at body temperature) is the prime determinant of postprandial lipemic responses. This is in accordance with previous observations that greater proportions of solid fat at body temperature in hardstocks were the main determinant of slower postprandial lipemic responses,^10,12,15,34,35^ most likely due to reduced rate of lipolysis and absorption. All three spreads had similar melt profiles but different proportions of lauric, myristic, palmitic and stearic acids. These novel findings suggest that neither the interesterification process nor fatty acid composition affects postprandial lipemia in healthy adults, when consumed as commercially-available product formulations.

Total serum TAG concentrations reflect both exogenous (chylomicron) and endogenous (VLDL) sources, as well as rates of entry and removal. To provide a more precise insight into potential differences in postprandial lipid metabolism between test fats and potential atherogenicity, we evaluated lipoprotein particle profiles. RO, richer in oleic acid, linoleic acid and alpha-linolenic acid, led to a more moderate increase in postprandial average HDL particle sizes and greater reductions in concentrations of extra-large, large and medium HDL particles, as well as ApoA1, compared with the spreads, which may be relevant to arterial health. The atherogenicity of different subclasses of HDL is currently the subject of debate,^36^ with larger HDL being associated with lower risk of CVD,^37^ but multivariable Mendelian randomization suggested that small (and medium) HDL particles were also protective against coronary artery disease.^38^ Further, reports from *in vitro* studies show that small HDL is more efficient at mediating cholesterol efflux from foam cell macrophages^39^ and are more potent than larger HDL at protecting LDL particles from metal-dependent and -independent oxidation.^40^ Therefore, this observed reduction in HDL particle size following the RO may confer favourable health effects protective of the vascular endothelium.^15,41^ In addition, the NMR analysis indicated that spreads led to reduced postprandial responses for ApoB and larger LDL particle sizes relative to RO. Small dense LDL particles are highly atherogenic.^42^ It has been reported previously that chronic intake of saturated fats produce LDL particles that are larger and more buoyant,^43^ although not in the presence of fasting hypertriglyceridemia;^44^ these findings corroborate previous observations that higher saturated fatty acid intakes in metabolically healthy adults may result in larger LDL particles in the latter postprandial period compared with a lower saturated fatty acid test meal. The key novel finding is that interesterification does not affect lipoprotein particle size or apolipoproteins.

There were no differences in postprandial insulin, c-peptide or glucose responses to the spreads or RO, but there were treatment effects on plasma NEFA concentrations, particularly during the latter half of the postprandial measurement period when circulating levels started to increase before the second meal. Circulating NEFA concentrations were higher following all the spreads relative to the RO comparator. One could speculate that the higher SFA-containing meals led to a higher fatty acid spillover into the circulation arising from lipoprotein lipase action on chylomicron TAG, which was clearly not modified by interesterification.^45^

Endothelial dysfunction, an early indicator of cardiovascular risk, was assessed using FMD. FMD is expected to decline following high-fat meals^19,46^ because of increased oxidative stress and inflammation arising from large increases in circulating TAG, leading to reduced nitric oxide bioavailability. Surprisingly, we found that FMD was not significantly impaired by high-fat meals, regardless of the nature of the test fat. This result contrasts with many older studies^19,46^ but agrees with a two RCTs that found no postprandial impairment in FMD following high-fat meals rich in saturated (butter), monounsaturated (olive oil/rapeseed oil) and n-6 polyunsaturated fatty acids (safflower oil),^47^ or palmolein and olive oil.^48^ Palmitic acid increases inflammation and impairs nitric oxide production from endothelial cells *in vitro*^49–51^ and appears to co-act with LPS in inducing macrophage inflammation.^52^ Therefore, it was hypothesised that IE spread would impair postprandial endothelial function to a lesser extent than non-IE spread due to its lower palmitic acid content. However, differences were small and not statistically significant. Furthermore, RO was low in palmitic acid yet FMD responses were similar to SB.

Since high-fat meals can increase markers of oxidative stress,^24,53,54^ it was expected that this would be reflected as increased NADPH oxidase activity after high-fat test meals, indicating greater superoxide production that would lead to scavenging of nitric oxide. There was no difference between the meals according to fatty acid unsaturation. This is in contrast to studies using other oxidative stress markers *e.g.* isoprostanes, finding that unsaturated fatty acids induces greater oxidative stress.^24,55,56^ The lack of impairment in postprandial FMD was consistent with the reduction in NADPH oxidase activity after all meals. The apparent postprandial reduction in NADPH oxidase activity may be the result of higher superoxide production following the 12 h overnight fast, a physiological response to fasting that would then be ameliorated following consumption of carbohydrate in the test meal muffins. This theory is supported by experimental data showing that NADPH oxidase activity was higher in pancreatic islets of 48 h fasted rats than fed rats.^57^ To our knowledge there are no other studies that have tested changes in neutrophil NADPH oxidase activity using this method following high-fat meals so further studies are needed to confirm this apparent postprandial reduction in superoxide production.

Although the postprandial response in inflammatory markers did not differ between spreads, there was a marked increase in serum GlycA following RO, with no parallel increase in serum IL-6 observed. To confirm that this was not due to interference from a MUFA peak in the NMR spectroscopy analysis, the analysis was repeated on serum GlycA : MUFA% and the same effect remained. This is a novel finding, which may be related to the high unsaturated fatty acid content of rapeseed oil. The lack of agreement between the two markers of postprandial inflammation, GlycA and IL-6, agrees with our previous work showing no correlation postprandially,^58^ although it should also be borne in mind that the indwelling cannula may itself induce a pronounced IL-6 response independently of high-fat meal consumption.^59^ The marked increase in serum GlycA concentrations contrasts with the modest increase observed after 50 g high-oleic sunflower oil in our previous study.^58^ Unlike high-oleic sunflower oil (5% linoleic acid), rapeseed oil contains 30% polyunsaturated fatty acids (10% α-linolenic acid and 20% linoleic acid), which may account for the different response in GlycA. The slower rates of lipolysis for the RO emulsion observed in the *in vitro* experiment might possibly promote production of acute phase proteins, possibly due to being absorbed lower down the small intestine, but this is an unlikely mechanism since plasma TAG responses were indistinguishable following RO compared to the spreads. The lack of agreement between the postprandial TAG data and the *in vitro* lipolysis experiment is unexpected, but may be related to differences in the matrix between the spreads and the RO oil. For example, although emulsion droplet size distributions did not differ between test fats, the emulsion droplet interface may have been altered by the presence of dairy proteins in the spreads dispersed in the 1% WPI aqueous phase, which may have increased the rate of lipolysis compared to RO.^60^

The investigation of biomarkers of endotoxemia following consumption of spreads and rapeseed oil is a novel direction of research. The healthy human gut microbiome is mainly populated by Gram-negative bacteria, whose outer membrane contains lipopolysaccharides (LPS), endotoxins. Following a fat-rich meal, endotoxins are transported into the bloodstream with the products of lipid digestion, thus potentially contributing to the initiation and continuation of postprandial inflammation.^32,61–63^ Higher blood concentrations of LBP and sCD14, markers of blood endotoxin exposure involved in the further inflammatory response,^31,64^ may be a link between postprandial lipemia and endothelial dysfunction.^65,66^ We found no significant postprandial increases in endotoxemia markers following spreads, but there was an elevation in sCD14 at 8 h following RO. Although this was not statistically significant using Bonferroni-adjusted pairwise comparisons, the trend is consistent with previous findings suggesting that high-fat meals enhanced transient postprandial endotoxemia,^30,32,63,67^ including a postprandial rise of sCD14.^30,32^ Further, these results agree with a study showing that 8-week rapeseed oil feeding in mice induced elevated levels of sCD14, but not increased inflammation measured by IL-6 in contrast to the response to a palm oil diet.^68^ We found that inflammation increased after all high-fat meals, but the IL-6 did not differ between fat types. However, the rise in GlycA concentrations was more pronounced followed RO, and then sharply decreased at 8 h, which may be linked to the buffering effects of sCD14.^68,69^

Sex differences in postprandial endotoxemia were observed; specifically, sCD14 decreased postprandially in males (mainly after RO and non-IE at 4 h) and slightly increased in females (driven by a marked increase after RO at 8 h). Sex differences in sCD14 responses have been reported previously. For example, associations between sCD14 and heart failure^70,71^ and CVD^71^ was only observed in females. In a cohort of antiretroviral therapy-treated people with human immunodeficiency virus, Looby *et al.* (2022) found higher sCD14 values among females, those in South Asia and sub-Saharan Africa, and older age groups.^72^ Modelling stratified by sex suggested that the trend of higher values of sCD14 among older age groups was driven by females.^72^ Altogether, these results show that this marker, sCD14, may be an important indicator of cardiovascular disease risk factor in middle-aged or older women.^73^

The strengths of the study include comparisons of functionally equivalent food products for real-life applicability, adequate statistical power to investigate differential responses by sex, postprandial testing for up to 8 h, a wash-out period long enough to avoid carry over effects, and inclusion of a range of novel mechanistic outcomes that complement the primary outcomes. Limitations include the fact that we cannot infer chronic effects of test fat consumption from this acute single-meal study, and similar effects in individuals at a high risk of cardiovascular disease cannot be assumed.

## Conclusions

An IE spread, with 17% palmitic acid content led to a similar degree of postprandial lipemia as a functionally matched non-IE spread with 28% palmitic acid content and a spreadable butter. Despite the striking similarity in postprandial TAG profiles following each of the test meals, there were differences in apolipoprotein/lipoprotein profiles between spreads and the comparator, rapeseed oil, namely a reduced elevation in ApoB, larger LDL particle sizes, and greater increases in ApoA1 and HDL particle sizes following spreads. No differences in endothelial function or oxidative stress were observed between test fats, but rapeseed oil provoked a greater increase in GlycA and an 8 h increase in a biomarker of endotoxin exposure relative to the spreads. The long-term implications of these differences in postprandial markers of cardiometabolic health are unclear but they point to a potentially neutral postprandial effect of a commercially available IE spread relative to functionally equivalent products made without IE fat in healthy adults. These findings pave the way for chronic dietary interventions using typically consumed fats to determine whether interesterification, by reducing saturated fatty acid content of foods, might be beneficial in reducing risk of cardiovascular diseases.

## Data availability

Data described in the manuscript and analytic code will be made available upon request to the corresponding author.

## Author contributions

WH conducted statistical analysis, wrote the initial manuscript draft, and finalised the submitted version. WH and SB conceptualised the study, acquired funding, supervised research activity planning and execution, designed the trials, interpreted data, and reviewed and edited subsequent versions. PG conducted the clinical trial and collected the data. AA was involved in running the clinical trial, conducted FMD measurements, analysed FMD images, and conducted statistical analysis. MD'A, AC, and MB were involved in running the clinical trial, and collected and analysed data, including fatty acids and NADPH oxidase. BL and AP analysed endotoxin data, and M-CM and FL analysed endotoxin data and assisted with scientific interpretation. LS and PW conducted the *in vitro* experiments. JB formulated the non-IE spread, provided test fat compositional data and provided advice on design of the dietary intervention. All authors commented on the initial manuscript draft and read and approved the final draft.

## Conflicts of interest

SB and WH are consultants to ZOE Ltd and SB also receives options in ZOE Ltd, but this is not related to the present study. JB is employed by a multinational agrochemical company (ADM) which manufactures vegetable oils including interesterified fats. M-CM received research funding from CNIEL, Sodiaal-Candia R&I and Danone Nutricia Research and has a research partnership with ITERG, which are not related to the present study. All other authors declared they had no conflicts of interest.

## Supplementary Material

FO-015-D3FO05324E-s001
