# Supplementary material for: Postprandial lipid and vascular responses following consumption of a commercially-relevant interesterified palmitic acid-rich spread in comparison to functionally-equivalent non-interesterified spread and spreadable butter: a randomised controlled trial in healthy adults
Source: Food Funct. 2024 Feb 15;15(5):2733–50. doi: 10.1039/d3fo05324e (PMC10911404; doi:10.1039/d3fo05324e)

Electronic Supplementary Material (ESI) for Food & Function.
This journal is © The Royal Society of Chemistry 2024

February 8th, 2024

Supplementary material: Postprandial lipid and vascular responses following consumption of a commercially-relevant interesterified palmitic acid-rich spread in comparison to functionally-equivalent non-interesterified spread and spreadable butter: a randomised controlled trial in healthy adults (Hall et al.)

	Page
Supplementary method for analysis of neutrophil NADPH oxidase activity	3
Supplementary Table 1 Incremental area under the curve (0-8 h) for postprandial serum lipoprotein subclass particle concentrations and lipoprotein size following an interesterified (IE) spread, non-IE spread, and spreadable butter (SB), with a reference rapeseed oil (RO).	5
Supplementary Figure 1. Emulsion droplet size distribution of emulsions prepared with IE spread, a non-IE spread, a spreadable butter (SB) and rapeseed oil (RO).	9
Supplementary Figure 2. Changes from baseline in plasma endotoxins presented by sex following an interesterified (IE) spread, non-IE spread, and spreadable butter (SB), with a reference rapeseed oil (RO)..	10
Supplementary Table 2 Postprandial incremental area under the curve (0-4 h and 0-8 h) for postprandial glucose, insulin, C-peptide and NEFA concentrations following an interesterified (IE) spread, non-IE spread,	12

and spreadable butter (SB), with a reference rapeseed oil (RO).	
Supplementary Figure 3. Lipolysis rates of emulsions prepared with IE spread, a non-IE spread, a spreadable butter (SB) and rapeseed oil (RO).	14

Supplementary method for analysis of neutrophil NADPH oxidase activity

Neutrophil NADPH oxidase activity was measured by the neutrophil oxidative burst assay and flow cytometry. Full details are in Supplementary material. All reagents were purchased from Sigma-Aldrich (Gillingham, UK) unless stated otherwise. Dihydrorhodamine123 (DHR123) was dissolved in dimethyl sulfoxide (DMSO) (2.5 mg/mL). Phorbol 12-myristate 13-acetate (PMA) was diluted in DMSO (100 µg/mL). These were then further diluted with phosphate buffered saline with azide (PBA) (with 2.5 % bovine serum albumin and 0.2 % sodium azide) yielding the final concentration of the working dilution of DHR123 at 15 µg/mL and PMA at 300 ng/mL. Stock ammonium chloride solution (Pharm Lyse, BD Biosciences) was diluted 1 in 10 in deionised water to give a working solution. Cells were stained with anti-human CD45 Krome Orange antibody (BioLedgend, London, UK) and fixed in 1% v/v formaldehyde in PBA.

Heparinized peripheral venous blood samples were obtained at 0, 4, and 6 h and tested immediately after the time of collection. Three fluorescence-activated cell sorting (FACS) tubes were labelled as (1) blood only, (2) DHR resting, and (3) PMA stimulated. To all the tubes, 100 µL of blood was diluted 1:10 with PBA. Immediately, 25 µL of DHR123 was added to tube 2 and 3 and all tubes were incubated in a warm-water bath (37°C). After 15 minutes, 100 µL of PMA solution was added to tube 3 to stimulate neutrophils to produce superoxide and undergo the oxidative burst. All tubes were incubated at 37°C for another 15 minutes during which time DHR123 is thereby oxidised to rhodamine (RHO). This releases a green fluorescent signal at 585 nm when excited by a 488 nm laser which can be measured by flow cytometry. Following the incubation, the samples were centrifuged and washed. The samples were then stained with 5 µL of anti-human CD45 antibody and lysed with

ammonium chloride for 15 minutes in the dark. Finally, the samples were centrifuged and washed again, and fixed in 1% formalin, before analysis of reagent ‘blood only’, ‘DHR resting’, and ‘PMA stimulated’ FACS tubes on a CytoFLEX Flow Cytometer (Beckman Coulter, Miami, FL), recording 10 000 neutrophil events. Data were subsequently analysed using CytExpert Software (Beckman Coulter, Miami, FL). The typical location of the neutrophil population was identified by the scattergram of CD45 expression vs. side scatter and selected by gating. Gating of the neutrophils in this CD45 positive region excludes debris and other cells present in the samples. A histogram of rhodamine fluorescence was obtained for the cells in the gated region of the flow scatterplot for each of the samples. Fluorescence is quantified by mean peak channel fluorescence; a weak level or no fluorescence at all indicates a reduced level of ROS. The NADPH oxidase activity is expressed as an oxidative index of neutrophils which is the ratio of mean fluorescence of PMA stimulated to mean fluorescence of DHR resting sample.

Supplementary Table 1 Incremental area under the curve (0-8 h) for postprandial serum lipoprotein subclass particle concentrations and lipoprotein size following an interesterified (IE) spread, non-IE spread, and spreadable butter (SB), with a reference rapeseed oil (RO).

	Time	***0 h***	***2 h***	***4 h***	***6 h***	***8 h***	**Treatment effect *P* value**	**Time[1] *P* value**	**Treatment x Time *P* value**
**ApoB, mg/mL[2]**	*RO*	0.761 (0.718, 0.806)	0.791 (0.744, 0.841)	0.815 (0.760, 0.874)	0.819 (0.764, 0.877)	0.768 (0.718, 0.822)	<0.001[4]	<0.001	0.015
	IE	0.761 (0.720, 0.804)	0.787 (0.742, 0.834)	0.802 (0.757, 0.851)	0.792 (0.748, 0.838)	0.769 (0.723, 0.817)			
	Non-IE	0.762 (0.719, 0.806)	0.774 (0.730, 0.821)	0.799 (0.749, 0.853)	0.781 (0.733, 0.831)	0.767 (0.720, 0.818)			
	SB	0.771 (0.729, 0814)	0.785 (0.740, 0.833)	0.804 (0.756, 0.854)	0.798 (0.751, 0.848)	0.772 (0.725, 0.821)			
**ApoA1, mg/mL[2]**	*RO*	1.501 (1.449, 1.555)	1.511 (1.460, 1.565)	1.524 (1.469, 1.580)	1.504 (1.455, 1.555)	1.573 (1.519, 1.630)	<0.015[4]	<0.001	NS
	IE	1.503 (1.447, 1.562)	1.515 (1.459, 1.573)	1.532 (1.478, 1.589)	1.529 (1.476, 1.583)	1.602 (1.523, 1.664)			
	Non-IE	1.519 (1.466, 1.574)	1.511 (1.459, 1.566)	1.547 (1.495, 1.600)	1.540 (1.489, 1.593)	1.604 (1.553, 1.657)			
	SB	1.502 (1.450, 1.556)	1.511 (1.457, 1.567)	1.532 (1.479, 1.587)	1.540 (1.486, 1.595)	1.589 (1.534, 1.646)			
**ApoB:ApoA1 ratio[2]**	*RO*	0.507 (0.475, 0.540)	0.527 (0.492, 0.564)	0.539 (0.500, 0.581)	0.547 (0.508, 0.589)	0.491 (0.454, 0.530)	<0.001[4]	<0.001	<0.001
	IE	0.506 (0.474, 0.540)	0.519 (0.486, 0.555)	0.524 (0.490, 0.560)	0.518 (0.485, 0.553)	0.480 (0.448, 0.514)			
	Non-IE	0.501 (0.467, 0.538)	0.512 (0.477, 0.550)	0.517 (0.479, 0.557)	0.507 (0.472, 0.544)	0.478 (0.445, 0.514)			
	SB	0.513 (0.481, 0.547)	0.519 (0.486, 0.556)	0.525 (0.490, 0.562)	0.518 (0.487, 0.552)	0.486 (0.453, 0.520)			
**XL-HDL-P concentration, μmol/L[3]**	*RO*	0.452 (0.389, 0.520)	0.411 (0.345, 0.483)	0.435 (0.365, 0.510)	0.488 (0.416, 0.566)	0.515 (0.454, 0.580)	<0.001[5]	<0.001	<0.005
	IE	0.462 (0.399, 0.529)	0.419 (0.350, 0.493)	0.472 (0.408, 0.540)	0.538 (0.477, 0.603)	0.548 (0.480, 0.619)			
	Non-IE	0.454 (0.385, 0.528)	0.428 (0.354, 0.508)	0.487 (0.416, 0.564)	0.567 (0.508, 0.630)	0.566 (0.506, 0.630)			
	SB	0.431 (0.358, 0.511)	0.407 (0.333, 0.489)	0.458 (0.383, 0.540)	0.550 (0.484, 0.620)	0.543 (0.476, 0.614)			
**L-HDL-P concentration, μmol/L[3]**	*RO*	1.125 (0.940, 1.327)	1.079 (0.892, 1.284)	1.062 (0.850, 1.299)	1.018 (0.803, 1.259)	1.338 (1.109, 1.590)	<0.001[5]	<0.001	0.006
	IE	1.146 (0.957, 1.351)	1.128 (0.941, 1.332)	1.176 (0.982, 1.387)	1.225 (1.023, 1.445)	1.500 (1.291, 1.725)			
	Non-IE	1.183 (0.992, 1.390)	1.150 (0.959, 1.358)	1.213 (1.009, 1.436)	1.288 (1.096, 1.495)	1.507 (1.306, 1.723)			
	SB	1.126 (0.942, 1.326)	1.106 (0.919, 1.310)	1.156 (0.962, 1.367)	1.247 (1.061, 1.448)	1.440 (1.239, 1.656)			
**M-HDL-P concentration, μmol/L**	*RO*	2.002 (1.907, 2.096)	1.955 (1.865, 2.045)	1.998 (1.890, 2.107)	1.904 (1.781, 2.028)	2.141 (2.036, 2.246)	0.001[5]	<0.001	0.091
	IE	2.005 (1.906, 2.105)	1.971 (1.872, 2.070)	2.019 (1.927, 2.112)	2.018 (1.922, 2.113)	2.233 (2.132, 2.334)			
	Non-IE	2.038 (1.933, 2.142)	1.952 (1.856, 2.048)	2.023 (1.928, 2.118)	2.022 (1.923, 2.122)	2.207 (2.110, 2.305)			
	SB	1.987 (1.905, 2.070)	1.955 (1.867, 2.042)	1.993 (1.899, 2.086)	2.010 (1.916, 2.104)	2.183 (2.085, 2.282)			
**S-HDL-P**	*RO*	4.800 (4.637, 4.963)	4.758 (4.604, 4.912)	4.745 (4.567, 4.924)	4.522 (4.358, 4.687)	4.710 (4.535, 4.886)	0.017[5]	<0.001	NS

	Time	***0 h***	***2 h***	***4 h***	***6 h***	***8 h***	**Treatment effect *P* value**	**Time[1] *P* value**	**Treatment x Time *P* value**
**concentration, µmol/L**	IE	4.823 (4.684, 4.963)	4.791 (4.640, 4.943)	4.747 (4.603, 4.891)	4.570 (4.421, 4.718)	4.700 (4.548, 4.852)			
	Non-IE	4.835 (4.692, 4.978)	4.709 (4.576, 4.843)	4.690 (4.563, 4.817)	4.510 (4.367, 4.652)	4.668 (4.518, 4.817)			
	SB	4.798 (4.640, 4.956)	4.770 (4.612, 4.929)	4.703 (4.534, 4.873)	4.534 (4.371, 4.698)	4.675 (4.502, 4.848)			
**HDL-P size, nm**	*RO*	10.01 (9.92, 10.09)	9.97 (9.88, 10.07)	10.00 (9.90, 10.09)	10.04 (9.95, 10.14)	10.11 (10.03, 10.20)	<0.001[5]	<0.001	<0.001
	IE	10.00 (9.92, 10.09)	9.99 (9.90, 10.07)	10.03 (9.94, 10.12)	10.106 (10.02, 10.19)	10.16 (10.08, 10.25)			
	Non-IE	10.02 (9.93, 10.10)	10.01 (9.92, 10.10)	10.06 (9.98, 10.15)	10.14 (10.06, 10.22)	10.18 (10.10, 10.26)			
	SB	10.00 (9.91, 10.09)	9.98 (9.89, 10.08)	10.04 (9.94, 10.13)	10.12 (10.03, 10.21)	10.16 (10.07, 10.24)			
**L-LDL-P concentration, umol/L[2]**	*RO*	0.156 (0.147, 0.166)	0.158 (0.149, 0.168)	0.154 (0.144, 0.164)	0.138 (0.129, 0.148)	0.150 (0.140, 0.162)	0.035[6]	<0.001	NS
	IE	0.158 (0.149, 0.168)	0.160 (0.151, 0.170)	0.154 (0.146, 0.163)	0.142 (0.134, 0.151)	0.155 (0.146, 0.165)			
	Non-IE	0.159 (0.151, 0.168)	0.157 (0.148, 0.167	0.153 (0.144, 0.163)	0.140 (0.131, 0.150)	0.151 (0.142, 0.161)			
	SB	0.160 (0.151, 0.170)	0.159 (0.150, 0.169)	0.155 (0.146, 0.164)	0.141 (0.132, 0.152)	0.151 (0.142, 0.161)			
**S-LDL-P concentration, umol/L[2]**	*RO*	0.149 (0.140, 0.159)	0.157 (0.147, 0.167)	0.150 (0.140, 0.161)	0.135 (0.126, 0.145)	0.144 (0.134, 0.154)	0.001[6]	<0.001	NS
	IE	0.152 (0.143, 0.162)	0.158 (0.148, 0.168)	0.149 (0.141, 0.159)	0.134 (0.126, 0.143)	0.144 (0.135, 0.154)			
	Non-IE	0.152 (0.144, 0.161)	0.154 (0.145, 0.163)	0.148 (0.139, 0.157)	0.132 (0.123, 0.141)	0.141 (0.131, 0.150)			
	SB	0.154 (0.144, 0.164)	0.157 (0.148, 0.167)	0.150 (0.141, 0.159)	0.135 (0.125, 0.144)	0.141 (0.132, 0.151)			
**LDL-P size, nm**	*RO*	23.53 (23.50, 23.56)	23.45 (23.42, 23.47)	23.48 (23.45, 23.52)	23.51 (23.46, 23.52)	23.54 (23.46, 23.56)	<0.001	<0.001	<0.001
	IE	23.52 (23.50, 23.54)	23.46 (23.43, 23.48)	23.51 (23.48, 23.48)	23.58 (23.54, 23.61)	23.59 (23.56, 23.62)			
	Non-IE	23.53 (23.50, 23.55)	23.48 (23.45, 23.50)	23.52 (23.49, 23.55)	23.60 (23.57, 23.633)	23.60 (23.57, 23.63)			
	SB	23.52 (23.49, 23.55)	23.45 (23.42, 23.48)	23.50 (23.47, 23.54)	23.57 (23.54, 23.61)	23.58 (23.55, 23,54)			
**XXL-VLDL/ chylomicron-P concentration (nmol/L)[2]**	*RO*	0.054 (0.041, 0.071)	0.084 (0.064, 0.109)	0.125 (0.096, 0.163)	0.221 (0.170, 0.288)	0.085 (0.065, 0.111)	NS	<0.001	NS
	IE	0.048 (0.036, 0.063)	0.073 (0.056, 0.095)	0.130 (0.100, 0.168)	0.188 (0.144, 0.244)	0.079 (0.060, 0.103)			
	Non-IE	0.050 (0.037, 0.066)	0.078 (0.060, 0.102)	0.134 (0.103, 0.175)	0.199 (0.153, 0.258)	0.090 (0.069, 0.118)			
	SB	0.048 (0.037, 0.064)	0.078 (0.059, 0.102)	0.119 (0.092, 0.155)	0.201 (0.155, 0.260)	0.086 (0.066, 0.112)			
**XL-VLDL particle concentrations (nmol/L)[2]**	*RO*	0.286 (0.202, 0.404)	0.364 (0.265, 0.501)	0.583 (0.432, 0.787)	0.959 (0.712, 1.291)	0.349 (0.251, 0.486)	NS	<0.001	NS
	IE	0.248 (0.179, 0.343)	0.361 (0.264, 0.493)	0.584 (0.430, 0.794)	0.863 (0.646, 1.153)	0.319 (0.230, 0.443)			
	Non-IE	0.289 (0.210, 0.397)	0.365 (0.268, 0.498)	0.653 (0.476, 0.894)	0.864 (0.639, 1.168)	0.421 (0.306, 0.581)			
	SB	0.258 (0.188, 0.354)	0.409 (0.303, 0.551)	0.550 (0.399, 0.757)	0.924 (0.688, 1.239)	0.391 (0.284, 0.538)			
**L-VLDL particle**	*RO*	2.244 (1.720, 2.927)	2.892 (2.242, 3.731)	4.053 (3.152, 5.212)	5.551 (4.304, 7.159)	2.486 (1.876, 3.294)	NS	<0.001	NS

	Time	***0 h***	***2 h***	***4 h***	***6 h***	***8 h***	**Treatment effect *P* value**	**Time[1] *P* value**	**Treatment x Time *P* value**
**concentrations (nmol/L)[2]**	IE	2.205 (1.717, 2,830)	2.826 (2.209, 3.615)	3.931 (3.098, 4.989)	5.137 (4.031, 6.547)	2.290 (1.753, 2.992)			
	Non-IE	2.203 (1.710, 2.837)	2.724 (2.120, 3.501)	4.053 (3.128, 5.252)	4.857 (3.754, 6.284)	2.935 (2.223, 3.875)			
	SB	2.156 (1.676, 2.774)	2.832 (2.188, 3.665)	4.012 (3.118, 5.161)	5.318 (4.149, 6.817)	2.832 (2.205, 3.637)			
**M-VLDL particle concentrations (nmol/L)[2]**	*RO*	11.22 (9.34, 13.47)	13.19 (11.03, 15.79)	15.32 (12.74, 18.42)	18.44 (15.28, 22.25)	10.80 (8.85, 13.17)	NS	<0.001	NS
	IE	11.26 (9.41, 13.48)	13.01 (10.92, 15.50)	15.66 (13.16, 18.63)	17.56 (14.65, 21.03)	10.80 (8.99, 12.96)			
	Non-IE	11.14 (9.29, 13.36)	12.66 (10.59, 15.13)	15.47 (12.87, 18.61)	16.96 (14.07, 20.44)	11.76 (9.65, 14.33)			
	SB	11.40 (9.56, 13.58)	12.62 (10.58, 15.07)	15.18 (12.70, 18.14)	17.97 (15.04, 21.47)	11.92 (9.93, 14.29)			
**S-VLDL particle concentrations (nmol/L)[2]**	*RO*	22.70 (20.13, 25.59)	24.85 (22.07, 27.98)	26.66 (23.66, 30.05)	27.41 (24.28, 30.94)	22.37 (19.75, 25.35)	NS	<0.001	NS
	IE	23.45 (20.87, 26.35)	25.06 (22.35, 28.10)	26.77 (23.91, 29.99)	27.08 (24.11, 30.42)	22.61 (20.14, 25.37)			
	Non-IE	23.10 (20.51, 26.03)	24.19 (21.51, 27.19)	26.27 (23.33, 29.58)	26.25 (23.27, 29.61)	23.26 (20.56, 26.33)			
	SB	23.57 (21.01, 26.46)	24.76 (22.06, 27.79)	26.53 (23.63, 29.77)	26.93 (23.96, 30.26)	23.27 (20.69, 26.18)			
**VLDL particle size (nm)**	*RO*	36.08 (35.64. 36.52)	36.66 (26.22, 27.09)	37.13 (36.66, 37.60)	37.94 (37.45, 38.43)	36.15 (35.64, 36.65)	NS	<0.001	NS
	IE	36.03 (35.59, 36.46)	36.54 (36.11, 36.74)	37.17 (36.74, 37.60)	37.68 (37.23, 38.14)	36.12 (35.67, 36.57)			
	Non-IE	36.01 (35.57, 36.44)	36.52 (36.09, 36.94)	37.18 (36.72, 37.64)	37.63 (37.15, 38.10)	36.42 (35.94, 36.90)			
	SB	36.04 (35.61, 36.46)	36.50 (36.07, 36.92)	37.09 (36.65, 37.53)	37.81 (37.36, 38.26)	36.41 (35.96, 36.87)			

Estimated marginal means (95% CI) at 0, 2, 4, 6 and 8 h, n=48. Data analysed using a linear mixed model with postprandial response as dependent variable; treatment, time, period, treatment * time and treatment * period as fixed effects; baseline values as covariate; subject as random effect; and post hoc pairwise comparisons adjusted for multiple comparisons using Bonferroni correction. [1]Excluding T0 from analysis, which is included as a covariate. [2] Analysis conducted on natural log transformed data. Geometric means with 95% CI shown. [3] Analysis conducted on square root transformed data.

[4]Apo B was significantly higher postprandially following RO compared with non-IE and SB, ApoA1 was significantly lower following RO compared with IE and SB, and ApoB:ApoA1 ratio was significantly higher postprandially following RO compared with IE, non-IE and SB (post hoc Bonferroni-adjusted pairwise comparison *P* <0.005 for all).

[5]XL-HDL and L-HDL particle concentrations were significantly lower postprandially following RO compared with IE, non-IE and SB, and M-HDL particle concentrations were significantly lower postprandially following RO compared with IE and SB (post hoc Bonferroni-adjusted pairwise comparison *P* <0.001 to <0.05). S-HDL particle concentrations were significantly higher postprandially following RO compared with non-IE (post hoc Bonferroni-adjusted pairwise

comparison P <0.05). Average HDL particle size was significantly smaller following RO compared with IE, non-IE and SB (post hoc Bonferroni-adjusted pairwise comparison P <0.001)

[6]Post hoc Bonferroni-adjusted pairwise comparisons were not statistically significant between treatments for L-LDL particle concentrations. S-LDL particle concentrations were significantly higher postprandially following RO compared with non-IE and SB (post hoc Bonferroni-adjusted pairwise comparison P <0.005 and <0.05 respectively). Average LDL particle size was significantly smaller following RO compared with IE, non-IE and SB (post hoc Bonferroni-adjusted pairwise comparison P <0.001), and following SB compared with non-IE (post hoc Bonferroni-adjusted pairwise comparison P <0.05).

There were significant treatment * period interactions for ApoB:ApoA1 ratio (P<0.001), S-LDL-P (P<0.001), LDL size (P<0.001), XXL-VLDL-P (P<0.05), XL-VLDL-P (P<0.05), L-VLDL-P (P<0.001), M-VLDL-P (P<0.01), S-VLDL-P (P=0.001).

IE: interesterified; SB: spreadable butter; S: small; M: medium; L: large; P: particle; XL: extra large; XXL: extra extra large.

Supplementary Figure 1. Emulsion droplet size distribution of emulsions prepared with IE spread, a non-IE spread, a spreadable butter (SB) and rapeseed oil (RO).

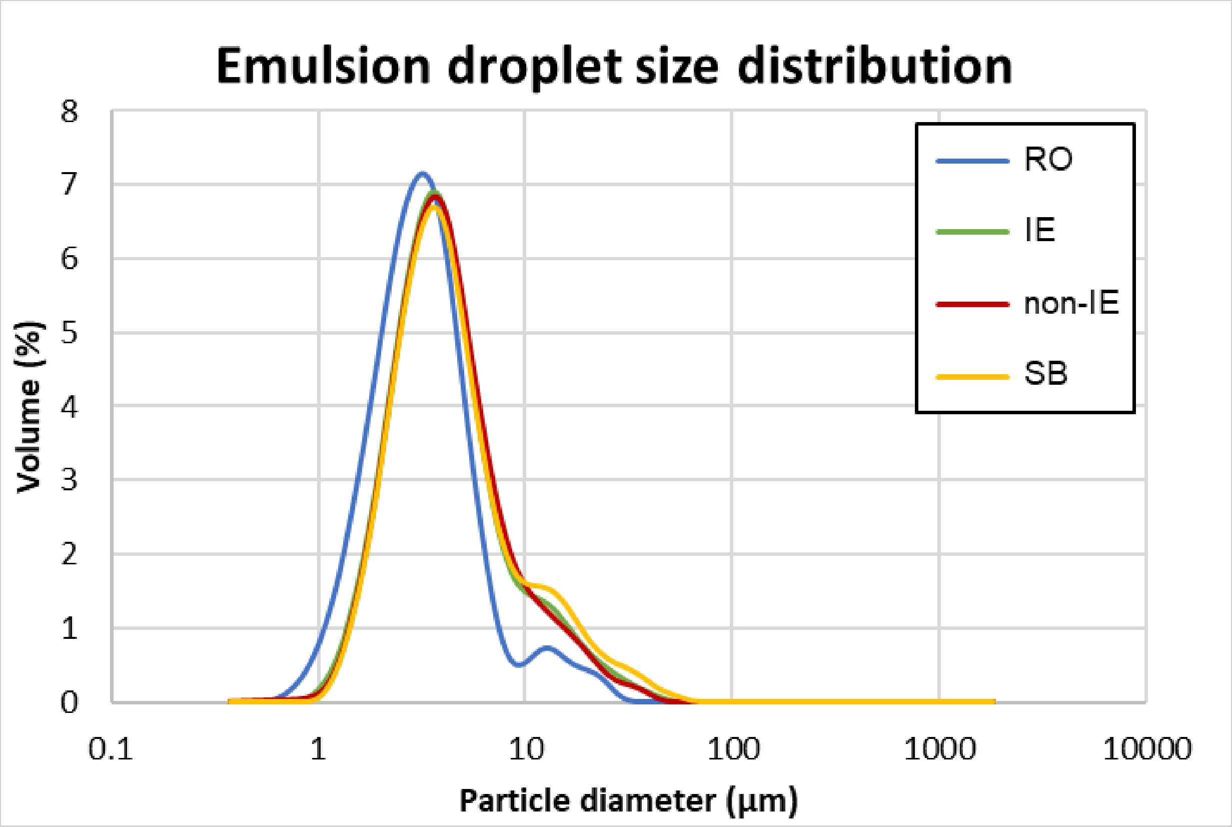

Supplementary Figure 2. Changes from baseline in plasma endotoxins presented by sex following high-fat test meals.

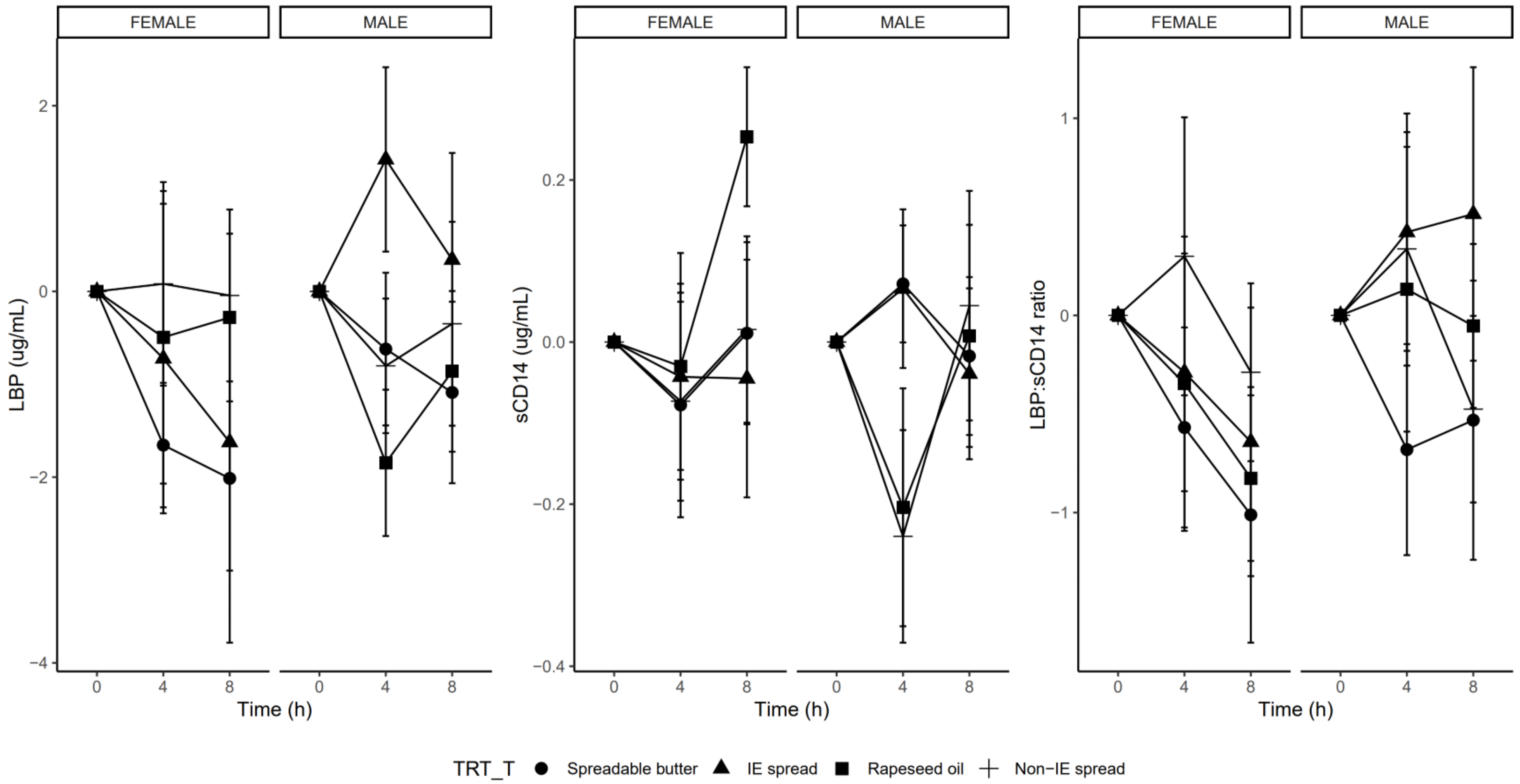

Changes from baseline in plasma lipopolysaccharide-binding protein (LBP) concentrations, soluble cluster of differentiation 14 (sCD14) concentrations, and LBP:sCD14 ratio (n=31-40 per treatment/time point) concentrations following a test meal containing 50 g fat from 3 different spreads (one commercially available containing interesterified (IE) palm oil fractions, palm kernel oil and rapeseed oil; one a non-IE equivalent from mid-fraction palm oil, palm kernel oil, and rapeseed oil; and the other a spreadable butter made with butter and rapeseed oil) relative to a reference rapeseed oil. Data are means with standard errors. Comparison of test fats by linear mixed-model analysis (dependent variable postprandial values, fixed factors of treatment, time, period, sex, treatment × time interaction, treatment × period interaction; treatment × sex; random effect participant; covariate baseline) showed no significant treatment or time effects. There was a significant sex effect (*P* =0.031) for sCD14, but no treatment × sex interactions; there was a tendency for sCD14 to decrease postprandially in males and increase in females. Females: IE n=19, non-IE n=19, SB n=20, RO n=16. Males: IE n=21, non-IE n=19, SB n=18, RO n=17.

Supplementary Table 2 Postprandial incremental area under the curve (0-4 h and 0-8 h) for postprandial glucose, insulin, C-peptide and NEFA concentrations following an interesterified (IE) spread, non-IE spread, and spreadable butter (SB), with a reference rapeseed oil (RO).

Outcome	Meal	iAUC (4h)	iAUC (8h)
Glucose (mmol/L.h)	SB	0.00 (-0.86, 0.85)	3.86 (2.50, 5.22)
	IE	-0.37 (-1.21, 0.48)	4.15 (2.78, 5.52)
	Non-IE	0.02 (-0.69, 0.73)	3.96 (2.64, 5.27)
	RO	0.44 (-0.42, 1.3)	4.06 (2.64, 5.48)
	Treatment effect (*P* value)	0.243	0.947
	Sex effect (*P* value)	0.149	0.519
	Treatment x sex interaction (*P* value)	0.097	0.825
Insulin (mIU/L.h) [1]	SB	79.0 (65.2, 95.6)	155.7 (132.1, 183.5)
	IE	85.0 (71.6, 100.7)	165.2 (140.2, 194.8)
	Non- IE	78.6 (66.0, 93.8)	152.9 (130.1, 179.9)
	RO	80.6 (68.2, 95.2)	151.4 (130.1, 176.1)
	Treatment effect (*P* value)	0.417	0.223
	Sex effect (*P* value)	0.125	0.167
	Treatment x sex interaction (*P* value)	0.944	0.669
C-peptide (ug/L.h) [1]	SB	9.69 (8.60, 10.92)	15.35 (12.21, 19.31)
	IE	9.91 (8.92, 11.02)	17.63 (15.81, 19.69)
	Non- IE	9.88 (8.87, 11.00)	16.96 (15.23, 18.90)
	RO	9.95 (8.97, 11.05)	16.98 (15.22, 18.96)
	Treatment effect (*P* value)	0.935	0.325
	Sex effect (*P* value)	0.075	0.053
	Treatment x sex interaction (*P* value)	0.761	0.340
NEFA (mmol/L.h)	SB	-1.27 (-1.38, -1.16)	-1.40 (-1.72, -1.09)
	IE	-1.28 (-1.38, -1.18)	-1.39 (-1.69, -1.09)
	Non- IE	-1.22 (-1.33, -1.10)	-1.28 (-1.60, -0.97)
	RO	-1.35 (-1.44, -1.26)	-1.69 (-2.00, -1.38)
	Treatment effect (*P* value)	0.014	0.002
	Sex effect (*P* value)	0.105	0.149
	Treatment x sex interaction (*P* value)	0.304	0.097

Values are estimated marginal geometric means, n=45. Data analysed on an intention-to-treat basis using a linear mixed model (fixed factors treatment, sex, period, treatment*sex, treatment*period; covariate was baseline; participant ID was a random factor). [1] Exponents of natural log transformed estimated marginal means. iAUC; incremental area under the curve; NEFA, non-esterified fatty acids. There were significant treatment effects for NEFA iAUC(0-4h) and iAUC(0-8h) (P = 0.014 and P = 0.002 respectively); post hoc tests with Bonferroni adjustment showed that the decrease in NEFA up to 4 hours was significantly greater following RO compared to non-IE (mean difference -0.13 mmol/L.h, 95% CI -0.25, -0.02, P = 0.015), and up to 8 hours was significantly greater following TO compared to SB, IE and non-IE (mean difference RO-SB was -0.29 mmol/L.h, 95% CI -0.57, -0.00, P = 0.048, mean difference RO-IE was -0.30 mmol/L.h, 95% CI -0.54, -0.06, P = 0.008, and mean difference RO-non-IE was -0.41 mmol/L.h, 95% CI -0.69, -0.13, P = 0.001) .

Supplementary Figure 3. Lipolysis rates of emulsions prepared with IE spread, a non-IE spread, a spreadable butter (SB) and rapeseed oil (RO).

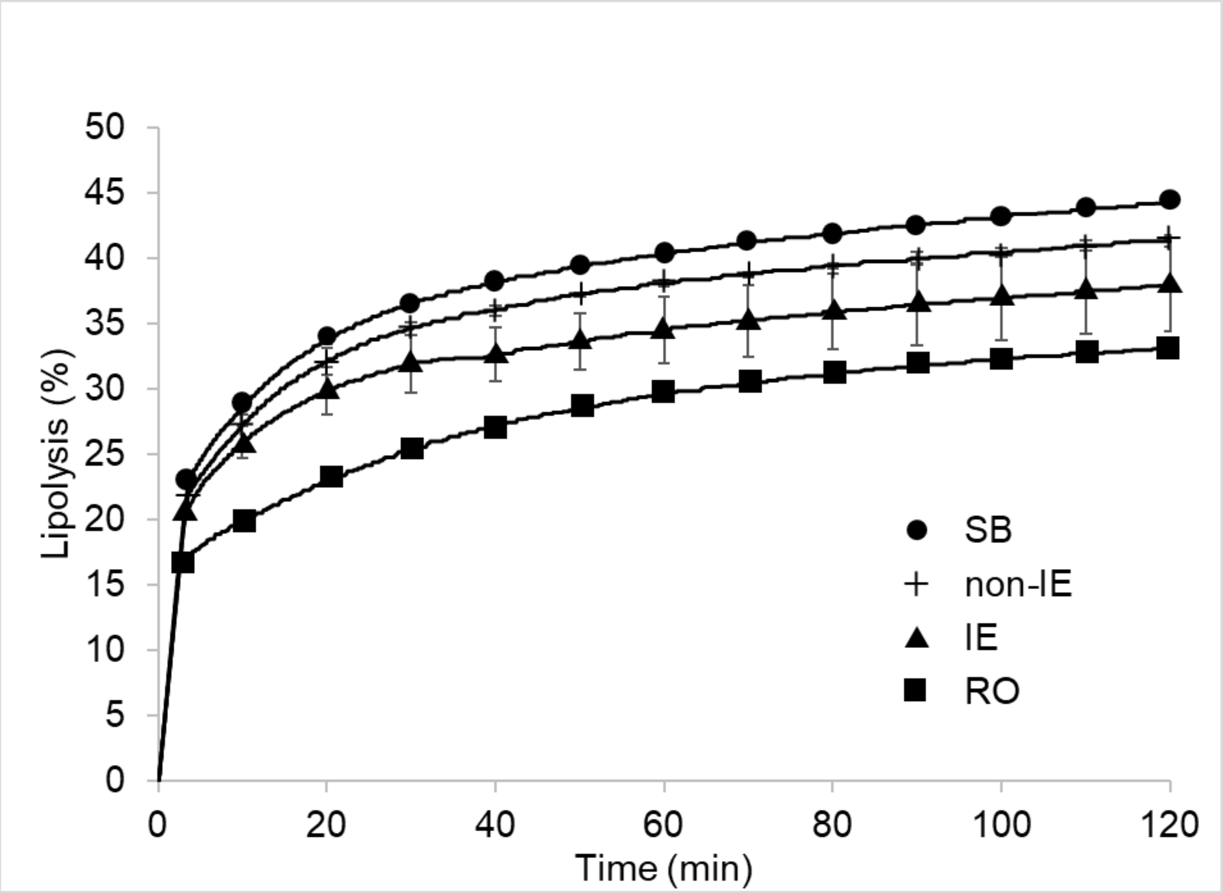

Supplement: FO-015-D3FO05324E-s001 [file FO-015-D3FO05324E-s001.pdf]
